# Genome structure and content of the rice root‐knot nematode (*Meloidogyne graminicola*)

**DOI:** 10.1002/ece3.6680

**Published:** 2020-09-13

**Authors:** Ngan Thi Phan, Julie Orjuela, Etienne G. J. Danchin, Christophe Klopp, Laetitia Perfus‐Barbeoch, Djampa K. Kozlowski, Georgios D. Koutsovoulos, Céline Lopez‐Roques, Olivier Bouchez, Margot Zahm, Guillaume Besnard, Stéphane Bellafiore

**Affiliations:** ^1^ IRD‐CIRAD‐University of Montpellier UMR Interactions Plantes Microorganismes Environnement (IPME) Montpellier France; ^2^ Institut Sophia Agrobiotech INRAE CNRS Université Côte d’Azur Sophia Antipolis France; ^3^ Plateforme BioInfo Genotoul UR875 INRAE Castanet‐Tolosan cedex France; ^4^ US 1426 GeT‐PlaGe Genotoul INRAE Castanet‐Tolosan France; ^5^ CNRS‐UPS‐IRD UMR5174 EDB Université Paul Sabatier Toulouse France

**Keywords:** cereals, horizontal gene transfer, pest, reference genome, root‐knot nematode, transposable element

## Abstract

Discovered in the 1960s, *Meloidogyne graminicola* is a root‐knot nematode species considered as a major threat to rice production. Yet, its origin, genomic structure, and intraspecific diversity are poorly understood. So far, such studies have been limited by the unavailability of a sufficiently complete and well‐assembled genome. In this study, using a combination of Oxford Nanopore Technologies and Illumina sequencing data, we generated a highly contiguous reference genome (283 scaffolds with an N50 length of 294 kb, totaling 41.5 Mb). The completeness scores of our assembly are among the highest currently published for *Meloidogyne* genomes. We predicted 10,284 protein‐coding genes spanning 75.5% of the genome. Among them, 67 are identified as possibly originating from horizontal gene transfers (mostly from bacteria), which supposedly contribute to nematode infection, nutrient processing, and plant defense manipulation. Besides, we detected 575 canonical transposable elements (TEs) belonging to seven orders and spanning 2.61% of the genome. These TEs might promote genomic plasticity putatively related to the evolution of *M. graminicola* parasitism. This high‐quality genome assembly constitutes a major improvement regarding previously available versions and represents a valuable molecular resource for future phylogenomic studies of *Meloidogyne* species. In particular, this will foster comparative genomic studies to trace back the evolutionary history of *M*. *graminicola* and its closest relatives.

## Graphical abstract

The genome of the rice root‐knot nematode (*Meloidogyne graminicola*) was assembled by combining short and long sequencing reads. Genes and transposable elements were annotated and then analyzed, revealing that lateral gene transfers from bacteria may have greatly contributed to the evolution of parasitism. This genome draft will foster comparative genomic studies to trace back the evolutionary history of *M. graminicola* and its closest relatives.

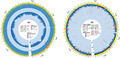

## 1 INTRODUCTION

*Meloidogyne graminicola*, commonly called the rice root‐knot nematode (rice RKN), is a prevalent pest at a global scale, causing severe damages to cereals (Dutta, 2012) and infecting more than 100 plant species (EPPO Global Database, 2019). This pest was first described in Louisiana (Golden & Birchfield, 1965) and Laos (Golden & Birchfield, 1968), before being found attacking several rice agrosystems (from upland to lowland, and irrigated to deep‐water fields) in many countries from America, Africa, Europe, and especially Asia. While Asia provides 90% of the global rice production, a 15% yield loss due to RKNs was estimated in this area, and this is probably an underestimate because of the lack of specific aboveground symptoms (Mantelin, Bellafiore, & Kyndt, 2017).

*Meloidogyne graminicola* is mainly reproducing through facultative meiotic parthenogenesis with a very short lifecycle (Narasimhamurthy et al., 2018). A freshly hatched juvenile can develop into an adult female laying 250–300 eggs after only 25–28 days. Such reproductive abilities may explain its rapid population increase and spread. For instance, in northern Italy, where this pest was recently detected, the total infected area has increased by approximately fivefold in just one year (from 19 to 90 ha in 2016–2017; EPPO Global Database, 2019). This nematode is therefore classified as a quarantine pest in several countries (e.g., Brazil, Madagascar, China; EPPO Global Database, 2019) and was added recently to the EPPO Alert List in Europe (Fanelli et al., 2017). Despite the huge impact of *M. graminicola* on agriculture worldwide, its evolutionary history and adaptive behavior in variable environments are still poorly documented. Therefore, control of this pathogen remains limited.

Root‐knot nematode species (RKNs; *Meloidogyne* spp.) exhibit a striking diversity of reproductive modes, chromosome counts, and hosts (Castagnone‐Sereno, Danchin, Perfus‐Barbeoch, & Abad, 2013). Those with obligate sexual reproduction have fewer chromosomes and a narrow host spectrum [e.g., *M*. *spartinae,* *n* = 7 (Triantaphyllou, 1987)], compared to those with facultative sexual reproduction [e.g., *M. graminicola*, *M. hapla*, *M. chitwoodii*; *n* = 13–19 (Triantaphyllou, 1985)], which have a broader host range and larger geographic distribution. Curiously, the most damaging RKNs to worldwide agriculture, owing to the diversity of infected hosts and most extensive global distribution, are reproducing asexually by obligatory mitotic parthenogenesis (Castagnone‐Sereno & Danchin, 2014). These species are polyploid with numerous chromosomes [e.g., *M. javanica,* 3n = 42–48 (Triantaphyllou, 1985)]. During the last fifteen years, advances in next‐generation genome sequencing have provided new insights into the considerable diversity and life history of plant‐parasitic nematodes (PPNs), particularly RKNs (Abad et al., 2008; Opperman et al., 2008). According to phylogenetic studies based on nuclear ribosomal DNA (nrDNA), RKNs can be classified in three main clades (De Ley et al., 1999), with most of the knowledge recently accumulated on species belonging to Clade I (e.g., *M. incognita*, *M. floridensis, M. javanica*, *M*. *arenaria,* and *M. enterolobii*) and Clade II (e.g., *M. hapla*). Comparative genomics on some mitotic parthenogenesis RKN species of Clade I provided relevant data on the origin and evolution of their polyploid genomes. Highly diverged genome copies and lack of recombination events were reported in these species, indicating hybrid origins and clonal reproduction (Blanc‐Mathieu et al., 2017; Koutsovoulos et al., 2019; Lunt, Kumar, Koutsovoulos, & Blaxter, 2014; Szitenberg et al., 2017). Besides, their genomes contain numerous transposable elements (TEs), while the meiotic facultative sexual diploid *M. hapla* (Clade II) does not show diverged genome copies and seems to have a lower TE load (Bird et al., 2009; Blanc‐Mathieu et al., 2017; Szitenberg et al., 2017). Horizontal gene transfers (HGTs) originating from bacteria and fungi have probably played an important role in the evolution of plant parasitism in RKNs, as well as in other nematode groups (Danchin & Rosso, 2012; Danchin et al., 2010; Haegeman, Jones, & Danchin, 2011). In RKNs, functional genes potentially acquired via HGT have been documented in *M*. *incognita*, *M*. *javanica*, *M*. *floridensis,* and *M*. *hapla* (Clades I and II) for proteins involved in plant cell‐wall degradation, nutrient processing, detoxification, and manipulation of plant defenses (Scholl, Thorne, McCarter, & Bird, 2003). Compared with other mitotic parthenogenetic and sexual RKNs, the diversity and genetic structure of facultative meiotic parthenogenetic species of Clade III remain, however, poorly understood. In *M*. *graminicola*, most of the genetic studies were based on mitochondrial DNA and nrDNA. These sequences revealed very low polymorphism and lack of phylogeographic signal among the isolates sampled at a global scale, suggesting a recent spread of this pathogen (Besnard et al., 2019). Divergent low‐copy nuclear homologous sequences were also found indicating either a potential hybrid origin or high heterozygosity in this species. These hypotheses, based on sporadic pieces of evidence, need to be better documented. Generating a high‐quality genome sequence of *M*. *graminicola* integrating close relatives is thus necessary for further comparative genomic analyses, especially to trace back their origin and global spread. Moreover, this will allow a better understanding of the impact of reproduction strategies and genome evolution in adaptive processes linked to different environmental conditions.

A first draft of the *M*. *graminicola* genome was released, with a genome assembly size of 35 Mb (Somvanshi, Tathode, Shukla, & Rao, 2018). However, the assembly was highly fragmented, totaling more than 4,300 contigs and an N50 length of 20 kb. In addition, compared with other RKN genomes, including the only other meiotic facultative sexual *M. hapla*, gene completeness (assessed on widely conserved single‐copy eukaryotic genes) was relatively low in this genome. For instance, respectively 84.27% and 73.60% of CEGMA and BUSCO eukaryotic genes were found in complete length in the *M. graminicola* genome versus respectively 93.55% and 87.40% for *M*. *hapla* (Koutsovoulos et al., 2019). This means that some genomic regions were probably not captured in the assembly or too fragmented. Therefore, the quality of this draft genome currently limits further sensitive studies such as comparative genomics of RKNs or population genomics studies at the species level. The reconstruction of the *M. graminicola* genome is challenged by two main features. Firstly, the *M. graminicola* genome is GC‐poor (GC content = 23.5%), which makes it extremely fragile and favors breaks during DNA extraction. Secondly, the genome is heterozygous (heterozygosity = ca. 2%), and its assembly is made difficult by the presence of divergent haplotypes, especially when using short reads (Besnard et al., 2019). For instance, some divergent homologous regions may be separately assembled, while others could be merged in a unique consensus sequence (Besnard et al., 2019).

To overcome these difficulties, we opted for a hybrid genome sequencing strategy, combining long reads (Oxford Nanopore Technologies, ONT) with high‐accuracy Illumina short reads to obtain a more complete and contiguous genome assembly. Genome assembly was performed with different software packages and strategies, and the one having the best biological and statistical metrics was finally selected. We annotated the genome for protein‐coding genes, TEs, and potential HGTs. Total DNA content of *M. graminicola* cells was also measured by flow cytometry to validate genome size. So far, this genome assembly is the most complete and contiguous available for *Meloidogyne* of Clade III, and this reference will assist a range of genetic, genomic, and phylogenetic studies to uncover the life history of *M. graminicola* and related RKNs.

## 2 MATERIALS AND METHODS

### 2.1 Nematode DNA extraction

The *M*. *graminicola* isolate Mg‐VN18 was isolated from rice roots collected in a high‐land field of the Lao Cai Province, Vietnam (Bellafiore et al., 2015). Mg‐VN18 was cultivated from a single juvenile on the root system of the susceptible rice cultivar IR64. Eggs and juveniles were extracted from roots 2 months after infection using a hypochlorite extraction method and a blender (McClure, Kruk, & Misaghi, 1973) with minor modifications from Bellafiore et al. (2015). Roots were treated for 15 min in 0.8% hypochlorite at room temperature to eliminate bacteria and fungi. After washing these nematodes carefully with water, the mixture was purified using discontinuous sucrose gradient as described in Schaad and Walker (1975) to remove potential remaining sources of DNA contaminants such as rice root tissues, bacteria, and fungi. After purification, the fresh eggs and juveniles were used directly for DNA extraction without freezing to avoid DNA fragmentation.

Getting high‐molecular‐weight DNA is a crucial step to benefit from the full potential of Oxford Nanopore Technologies (ONT) sequencing. Two different DNA extraction protocols were tested [i.e., protocol of Epicentre's MasterPure Complete DNA Purification Kit (Lucigen) and a modified phenol–chloroform‐based method (Sambrook, Fritsch, & Maniatis, 1989)]. The phenol protocol method yielding good‐quality DNA with an average fragment length of 39 kb for a total of 8.2 μg is suitable for ONT sequencing. Following this protocol, 260 µl of extraction buffer (0.1 M Tris, pH 8, 0.5 M NaCl, 50 mM EDTA, 1% SDS) and 40 µl of proteinase K (20 mg/ml; Qiagen) were added into the tube containing 0.1 ml of fresh eggs and juveniles. Nematodes were then crushed by twisting with an autoclaved micropestle for about 30 s. The solution was incubated at 55°C for 24 hr. Then, 10 µl of RNAse A (10 mg/ml; Qiagen) was added and the mix was incubated at room temperature for 50 min. Genomic DNA (gDNA) was recovered by a phenol–chloroform step (Sambrook et al., 1989). The chloroform‐free phase was treated with NH_4_OAC (for a final concentration of 0.75 M) before ethanol precipitation. To reduce DNA fragmentation, no freezing nor vortexing steps were performed. All the mixing steps were done by three meticulous tube inversions, and final gDNAs were stored at 4°C for less than one week before sequencing. For Illumina sequencing, gDNA was extracted following the manual of the Epicentre's MasterPure Complete DNA Purification Kit (Lucigen). For all gDNA samples, double‐stranded DNA concentration was assessed using the Qubit dsDNA HS Assay Kit (Life Technologies). DNA purity was checked using the NanoDrop (Thermo Fisher Scientific). Distribution and degradation of DNA fragment sizes were assessed using the Fragment analyzer (AATI) High Sensitivity DNA Fragment Analysis Kit (Thermo Fisher Scientific). DNA integrity was also checked by electrophoresis, loading 1 µl on a 1% agarose gel.

### 2.2 Whole‐genome sequencing, read processing, and *k*‐mer analysis

#### 2.2.1 Long‐read sequencing

Library preparation and sequencing were performed at the GeT‐PlaGe core facility, INRA Toulouse, according to the manufacturer's instructions “1D gDNA selecting for long reads (SQK‐LSK109).” Aiming at covering the *M*. *graminicola* genome at >70× with long reads, sequencing was done on one ONT flowcell. Genomic DNA was purified using AMPure XP beads (Beckman Coulter). Eight micrograms of purified DNA was sheared at 20 kb using the megaruptor system (Diagenode). A “one‐step” DNA damage repair + END‐repair + dA tail of double‐stranded DNA fragments was performed on 2 µg of DNA. Adapters were ligated to the library that was then loaded (0.03 pmol) onto an R9.4.1 revD flowcell. It was sequenced on the GridION instrument for 48 hr. Final reads were base‐called using Guppy v.1.8.5‐1 (Oxford Nanopore).

After sequencing, adapters of raw ONT reads were trimmed using Porechop (Wick, 2019). Only reads with a *Q*‐score value greater or equal to 7 were selected using NanoFilt v.1.1.0 (De Coster, D’Hert, Schultz, Cruts, & Van Broeckhoven, 2018). Minimap2 (Li, 2018) was used to map long reads to the *M*. *graminicola* mitogenome (GenBank no. HG529223), and Samtools Fasta ‐ f 0x4 (Li et al., 2009) was used to sort out long reads that mapped to this reference.

#### 2.2.2 Short‐read sequencing

High‐depth short‐read sequencing was performed at the GeT‐PlaGe core facility, INRA Toulouse. DNA‐seq libraries have been prepared according to the Illumina's protocol “TruSeq Nano DNA HT Library Prep Kit” (Illumina Sequencing Technology). Briefly, three micrograms of gDNA was fragmented by sonication. Then, DNA fragments were selected by size (mean insert size = approx. 380 bp) using SPB beads (kit beads), and then ligated to adaptors. Quality of libraries was assessed using a Fragment Analyzer (Advanced Analytical), and DNA quantity was measured by qPCR using the Kapa Library Quantification Kit (Roche). Sequencing was performed on an Illumina HiSeq‐3000 using a paired‐end read length of 2 x 150 bp with the Illumina HiSeq 3000 Reagent Kits.

Illumina raw reads were trimmed and cleaned from contamination. Firstly, the short reads were processed for quality control using FastQC (Andrews, 2010). Secondly, Skewer (Jiang, Lei, Ding, & Zhu, 2014) was used to trim reads considering a minimum quality score of 30 and a minimum read length of 51 bp. Thirdly, the trimmed reads were preassembled using Platanus (Kajitani et al., 2014). Subsequently, the preassembled contigs were blasted against the NCBI’s nucleotide (nt) database using Blastn (Altschul, Gish, Miller, Myers, & Lipman, 1990) for contamination screening on BlobTools (Kumar, Jones, Koutsovoulos, Clarke, & Blaxter, 2013; Laetsch & Blaxter, 2017). A group of preassembled contigs annotated as proteobacteria at low coverage (<10×) was considered as contaminants. Therefore, the reads that belonged to these contigs were removed from the pool of short reads, resulting in a cleaned Illumina dataset. The cleaned reads that aligned to the mitogenome of *M*. *graminicola* (GenBank no. HG529223) were also removed using Bowtie2 (Langmead & Salzberg, 2012). Finally, the reads were error‐corrected using Musket (Liu, Schroeder, & Schmidt, 2013).

Jellyfish (Marçais & Kingsford, 2011) was used to extract and count canonical *k*‐mers (*k* = 17, 21, 27, and 47 nucleotides) from cleaned Illumina reads. For each *k* value, GenomeScope (Vurture et al., 2017) was used to estimate haploid genome length, heterozygosity, and repeat content from the *k*‐mer counts. The parameter *MaxCov *was set at 900,000, as recommended by Mgwatyu, Stander, Ferreira, Williams, and Hesse (2020).

### 2.3 Quantification of nuclear DNA content

To assess the nuclear genome size of Mg‐VN18, two independent flow cytometry runs were done for five replicates, which were collected at different time points. Eggs and juveniles from each replicate were extracted and purified using the same method described above, then stored at −82°C. Besides, two species with known genome size, *Caenorhabditis elegans* strain Bristol N2 [200 Mb, diploid (The C. elegans Sequencing Consortium, 1998)] and *Drosophila* *melanogaster* Canton‐S strain [350 Mb, diploid (Bosco, Campbell, Leiva‐Neto, & Markow, 2007)], were used as internal standards. In each run, nucleus extraction, nucleus stain, and DNA content measurements were done using the same protocol as previously described (Blanc‐Mathieu et al., 2017; Perfus‐Barbeoch et al., 2014) for both samples and internal standards. In short, 0.1 ml of fresh eggs and juveniles was ground carefully for 7 min in 2 ml of the lysis buffer (1 mM KCl, 30 mM NaCl, 10 mM MgCl_2_, 0.2 mM EDTA, 30 mM Tris, 300 mM sucrose, 5 mM sodium butyrate, 0.1 mM PMSF, 0.5 mM DTT, 40 μl Igepal), and then, 8 ml suspension buffer (same as lysis buffer except for sucrose, 1.2 M, and without Igepal) was overlaid on top of lysis buffer. Subsequently, the tube was centrifuged to separate nuclei from other cell debris. After removing the supernatant, the pellet of nuclei was resuspended in 1 ml of staining buffer containing propidium iodide (final concentration of 75 μg/ml) and DNAse‐free RNAse (final concentration of 50 μg/ml) at 37°C for 30 min. Each sample was first measured independently and then mixed with standard controls in the same tube. Flow cytometry analysis was then performed using the LSR II/Fortessa (BD Biosciences) flow cytometer operated with the FACSDiva v.6.1.3 software (BD Biosciences). For each measurement, the fluorescence cytograms were analyzed on Kaluza v.1.2 (Beckman Coulter). For each species, fluorescent peaks corresponding to three phases of the cell cycle (G0/G1, S, and G2/M) were obtained (Ormerod, 2008). Only mean fluorescence intensity of the G0/G1 phase (first peak) was taken into account, and *M*. *graminicola* DNA content was then estimated using the following equation:

$$\text{Total DNA content of}\;M.graminicola\;\text{sample}=\frac{G0/\text{G1}\;\text{peak}\;\text{value}\;\text{of}\;\text{sample}\times \text{whole}\;\text{genome}\;\text{size}\;\text{of}\;\text{internal}\;\text{control}\;i}{G0/\text{G1}\;\text{peak}\;\text{value}\;\text{of}\;\text{internal}\;\text{control}\;i}$$

with *i* being either *C. elegans* or *D. melanogaster*.

### 2.4 Genome assembly, completeness assessment, and haplotigs purging

Five popular assemblers were first tested to assemble the *M*. *graminicola* genome: Flye v.2.4.1 (Kolmogorov, Yuan, Lin, & Pevzner, 2019), Ra v.0.2.1 (Vaser & Šikić, 2019), MaSuRCA v.3.2.4 (Zimin et al., 2013), Canu v.1.8 (Koren et al., 2017), and Miniasm v.2.2.16 (Li, 2016). Flye, Ra, Canu, and Miniasm use long reads only to build contigs, while MaSuRCA combines both long (ONT) and short (Illumina) reads. Subsequently, Racon (Vaser, Sovic, Nagarajan, & Sikic, 2017) and Pilon (Walker et al., 2014) were used to correct bases and homopolymer lengths. To scaffold the genome, a set of 66,396 transcripts (Petitot et al., 2016) was blasted to the genome assemblies. Then, the Perl script SCUBAT v.2 (Koutsovoulos, 2018) was used to identify transcripts that were split over multiple contigs. This information was then used to concatenate the contigs. After obtaining corrected and concatenated contigs, assembly statistics were computed using QUAST (Gurevich, Saveliev, Vyahhi, & Tesler, 2013) and compared. The genome completeness was assessed using both CEGMA [Core Eukaryotic Genes Mapping Approach (Parra, Bradnam, & Korf, 2007)] and BUSCO v.3 [Benchmarking Universal Single‐Copy Orthologs (Simão, Waterhouse, Ioannidis, Kriventseva, & Zdobnov, 2015)]. For CEGMA, the provided core set of 248 eukaryotic orthologs was used as a reference, and genes were predicted using default parameters (e.g., maximum intron length of 5 kb and gene flanks of 2 kb). For BUSCO, the provided nematoda dataset is not appropriate for RKNs because it contains orthologous genes of eight nematode species belonging to only three (2, 8, and 9) out of the 12 described nematoda clades (Megen et al., 2009) and no species from Clade 12, to which RKNs belong. Meanwhile, the eukaryotic dataset is a pool of single‐copy orthologs from 65 eukaryote species, including the nematoda dataset. Therefore, the “Eukaryota_odb9” library including 303 eukaryote single‐copy orthologs was preferred and used as the reference. The species‐specific trained parameters of the nematode species *C*. *elegans* were used for gene prediction and BUSCO was run in “‐long” mode for AUGUSTUS optimization. We used both the median length of scaffolds (N50) and genome completeness (i.e., the percentage of fully assembled conserved eukaryote genes) to select the best genome assembly for further analyses.

Heterozygous regions can severely complicate genome assembly with regions of higher heterozygosity being assembled separately, while regions of lower heterozygosity being collapsed in one consensus region. This may cause issues with genome size estimation, spurious annotation, variant discovery, or haplotype reconstruction. An ideal haploid representation (primary contigs) would consist of one allelic copy of all heterozygous regions in the two haplomes, as well as all hemizygous regions from both haplomes. Purge haplotigs (Roach, Schmidt, & Borneman, 2018) was used to identify contigs that were likely to be allelic contigs and retained only the primary contig. Briefly, in a first step, the program created a read‐depth histogram using the mapped long reads to the assembly. If the histogram shows only one read‐depth peak, there is no need to purge haplotigs because the entire genome contains collapsed haplotype contigs. Otherwise, if two peaks are observed, one being at half the coverage of the second, both allelic contigs and collapsed haplotype contigs are present in the assembly. For collapsed haplotypes, the reads from both alleles will map to the same contig, resulting in one read‐depth peak. In contrast, if the alleles are assembled as separate contigs, the reads will be split over the two contigs, resulting in another peak at half the read depth (“0.5 unit” read‐depth peak). The half read‐depth contigs will be assigned as suspect contigs (or supposedly uncollapsed contigs). In the second step, these suspect contigs are aligned against the entire genome to identify synteny with its allelic companion contig. Contigs with an alignment score greater than the cutoff (by default ≥70%) are marked for reassignment as haplotigs and removed from the assembly. In addition, the contigs with an abnormally low long‐read depth (≤10×) are likely to be assembly artifacts, while those with unusually high‐read depth (≥195×) are likely to be collapsed repeats, organellar DNA contigs, or contaminants. Such contigs were thus also removed from the rest of the assembly. Finally, the program will produce three FASTA format files: contigs reassigned as haplotigs, the abnormally covered contigs reassigned as artifacts, and the curated contigs that represent the haploid assembly.

The purged‐haplotigs genome (curated contigs) was then blasted to the NCBI nt database using Blastn (Altschul et al., 1990) for contamination screening on BlobTools (Kumar et al., 2013; Laetsch & Blaxter, 2017). Contigs with short‐read depth inferior to 100× showing highest similarity to non‐nematoda sequences were considered as potential contaminants and thus removed from the assembly.

To investigate the heterozygous regions on the genome, the short reads were mapped against the curated genome assembly to call single nucleotide variants (SNV) using TOGGLE’s configuration file *SNPdiscoveryPaired.config.txt* (Tranchant‐Dubreuil et al., 2018). The reads from the two divergent haplotype copies will map on a single collapsed region in the reference genome, resulting in heterozygous SNVs. SNV positions with mapping quality ≥30 and sequencing depth ≥10× were selected. The number of heterozygous variants per 10‐kb window was then calculated using BEDOPS (Neph et al., 2012). The above short‐read mapping file was also used to calculate short‐read depth per window using BEDtools *multicov* (Quinlan & Hall, 2010). Long reads were mapped onto the genome using Minimap2 (Li, 2018) to generate a long‐read mapping file. The mapping file was sorted using Samtools *sort* and used for the calculation of long‐read depth per genome window using BEDtools. GC content per sliding window of 1 kb was calculated using BEDtools *nuc* (Quinlan & Hall, 2010). The distribution of heterozygous variants, short‐read depth, long‐read depth, and GC content was shown on the genome scaffolds per 10‐kb sliding window using CIRCOS (http://circos.ca/).

### 2.5 Gene prediction, annotation, and detection of putative horizontal gene transfers

Protein‐coding genes were predicted with the MAKER v.2.31.9 genome annotation pipeline (Holt & Yandell, 2011). To improve homology search during the annotation process, low‐complexity regions, satellites, and simple sequence repeats (SSR) were soft‐masked with lower‐case letters in the genome using RepeatMasker v.4.0.7 (http://www.repeatmasker.org). A transcriptome of *M*. *graminicola* at juvenile stage (Petitot et al., 2016) was used as source of evidence for gene predictions. A de novo transcriptome assembly was obtained using Trinity v.2.5.1 (Grabherr et al., 2011). For a given locus of the Trinity output, only the contigs with the longest ORF were kept. Hisat2 v2.1 (Kim, Langmead, & Salzberg, 2015) and StringTie v.1.3.4 0 (Pertea et al., 2015) were used to obtain a guided assembly of transcripts. Finally, four datasets were thus used as references: (a) the available dataset of 66,396 ESTs (Petitot et al., 2016), (b) the longest transcripts among their isoforms assembled by Trinity, (c) the whole transcripts assembled by StringTie, and (d) the *EST_nematoda* UniProt database. MAKER was run in two steps. The first step was based on pieces of evidence from the transcriptomes (*est2genome*) and protein sequences from UniProt and Trembl databases (*protein2genome*). In the second step, MAKER predicted genes by reconciling evidence alignments and ab initio gene predictions using SNAP v.2013‐11‐29 (Korf, 2004). Functional annotation for predicted genes was done by searching homology to UniProt/Swiss‐Prot databases. In addition, InterProScan v.5.19‐58.0 (Zdobnov & Apweiler, 2001) was used to examine conserved protein domains, signatures, and motifs present in the predicted protein sequences. Gene sequences with annotation edit distance (AED) values of less than one with domain content were retained using the Perl script *quality_filter.pl* (Campbell, Holt, Moore, & Yandell, 2014). The higher the AED value was, the higher the sequence divergence was detected between the predicted protein and the sources of evidence. The statistics of the gene prediction and annotation were retrieved using the Python script Genome Annotation Generator *gag.py* (Hall, DeRego, & Geib, 2014). Further, to infer the completeness of the predicted protein‐coding genes, the BUSCO score was calculated using the parameters described above for the genomic sequence. The number of genes per sliding genome window of 10 kb was calculated using BEDOPS (Neph et al., 2012). Distribution and density of genes on genome scaffolds were visualized using CIRCOS.

The coding genes were then used to detect candidate horizontal gene transfers (HGTs) of nonmetazoa origin in the *M*. *graminicola* genome using Alienness (Rancurel, Legrand, & Danchin, 2017). Basically, Alienness identifies genes in *M*. *graminicola* that are substantially more similar to nonmetazoan than metazoan homologs. In a first step, all the predicted proteins were compared with the NCBI’s nr library using BLASTp with an E‐value threshold of 1E^−3^ and no filtering for low‐complexity regions. Because we were looking for genes of nonmetazoan origin in a metazoan, we selected “Metazoa” as taxonomic recipient group. To avoid self‐hits to RKNs and other related plant‐parasitic nematodes, we excluded the suborder “Tylenchina.” Besides Bacteria, two additional taxonomic groups—Viridiplantae and Fungi—were used to classify the potential donors. Then, based on the taxonomy identity and the E‐value for each blast hit, Alienness calculates an Alien Index (AI) for each query protein as following: AI = ln(best metazoan E‐value + 1E^−200^) − ln(best nonmetazoan E‐value + 1E^−200^). An AI > 0 indicates a better hit to the donor (nonmetazoan) than recipient (metazoan) taxa and a putative HGT of nonanimal origin. Higher AI represents a higher gap of E‐values between candidate donor and recipient and a more likely HGT. According to the 70% rule (Ku & Martin, 2016), all *M*. *graminicola* proteins returning an AI > 0 with a 70% identity to a putative donor were discarded from the rest of the analyses to eliminate possible assembly or annotation artifacts. As recommended by Rancurel et al. (2017), an AI threshold > 14 represents the right balance between recall and precision of the method, at least in RKNs. With an AI > 26, the accuracy (proportion of candidate genes supported as HGT by phylogenies) is even higher, but the recall rate is lower (Rancurel et al., 2017). Therefore, in our study, we used both values as thresholds to detect putative HGTs and highly likely HGTs. Location of these genes on the whole genome was finally represented using CIRCOS.

### 2.6 Annotation of transposable elements

The assembled genome of *M*. *graminicola* was finally used to investigate transposable elements (TEs) using the REPET metapipeline, which includes TEdenovo and TEannot (Flutre, Duprat, Feuillet, & Quesneville, 2011). The TE prediction and annotation protocols followed in this study are described in details in Koutsovoulos et al. (2019). In brief, all the unresolved regions (Ns) of the genome longer than 11 nucleotides were first removed. Then, genomic sequences shorter than the L99 (5,010 bp) were discarded. Remaining sequences were used as input for the TEdenovo pipeline to de novo build a TE consensus library. The obtained sequence library was then automatically filtered doing a minimal genome annotation with TEannot and only retaining consensuses with at least one full‐length copy (FLC) annotated on the genome. The filtered consensus TE library was then used in the TEannot pipeline to perform a full annotation of the whole *M*. *graminicola* genome. Finally, strict filters were applied to only retain annotations conform to two main criteria: (a) Conserved TE annotations must be classified as retrotransposons or DNA transposons and be longer than 250 bp; and (b) TE copies must share 85% identity with their consensus and cover more than 33% of its length. Distribution of TEs on the genome was visualized using CIRCOS.

## 3 RESULTS

### 3.1 Whole‐genome sequence and total DNA content of *M. graminicola*

In total, 3.9 Gb of raw reads was produced by the Oxford Nanopore Technology (N50 length = 8.9 kb), while Illumina sequencing technology generated 122 million reads with a total volume of 17.4 Gb. After cleaning, 3.5 Gb of long reads with an N50 length of 9.4 kb and 87 million short reads (11.98 Gb) were retained (Table S1). The *k*‐mer analysis on cleaned short reads allowed us to estimate the haploid genome length at different *k* values, from 41.1 to 41.6 Mb with average heterozygosity varying from 1.69% to 1.90% (Table S2). In contrast, the repeat content of the genome dramatically depended on the *k* value used, although the highest values (*k* = 27 and 47) rendered similar results (7.8 Mb; Table S2).

**Figure 1:**
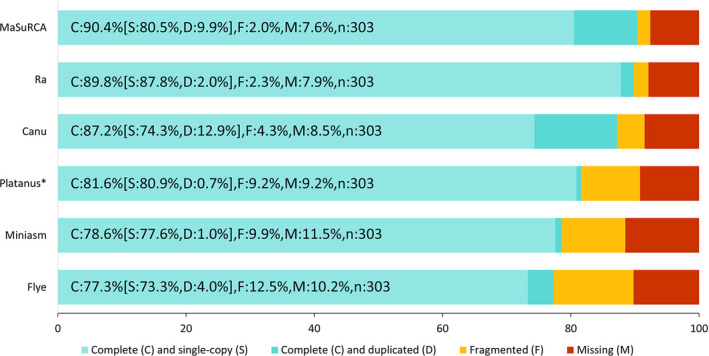
BUSCO completeness of genome assemblies generated with different assemblers. Five assemblies were generated in our study and are compared with the published assembly (Somvanshi et al., 2018) that was reconstructed with Platanus (indicated by the asterisk)

The cleaned long and short reads were used for the genome assembly. After polishing, the assembly length obtained with the five methods ranged from 39 (Ra) to 56 Mb (Canu) with a GC content of 23%–24% (Table S3). The contig‐scaffolding process allowed reducing the number of contigs and increasing the N50 length with no effect on genome GC content and CEGMA score, except for Miniasm (Table S3). Among the five methods, the Miniasm assembler returned the lowest number of contigs and the longest contig (~2 Mb), as well as the largest N50 length (425 kb). However, the completeness measured on eukaryotic BUSCO genes was the second worst (78.6%; Figure 1), casting doubt on the per‐base quality of the assembly. The three assemblies MaSuRCA, Ra, and Canu returned a BUSCO completeness score greater than 87% and were then selected for further steps (Figure 1).

**Figure 2:**
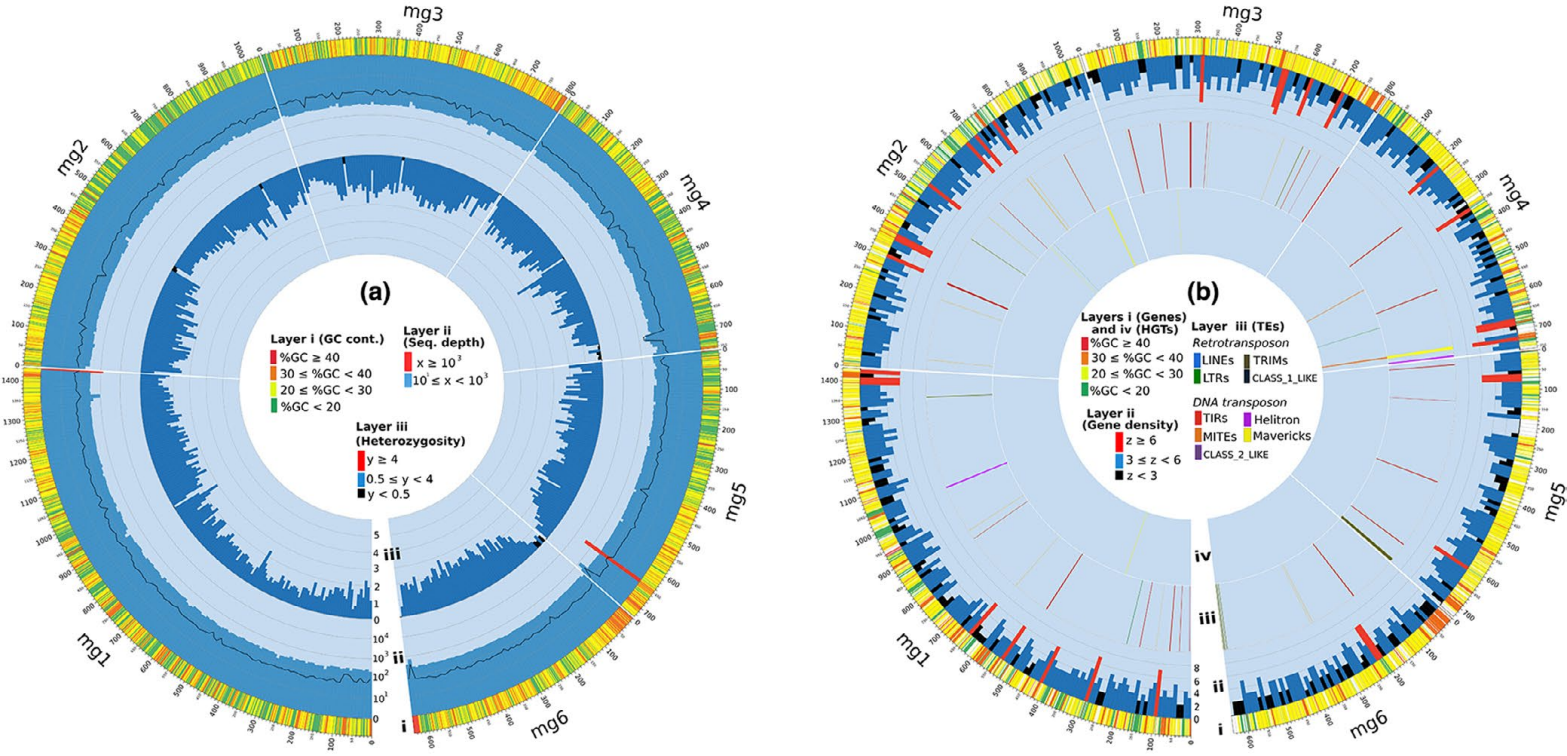
Genomic features along the six longest scaffolds (mg1 to mg6) with total size of 5.4 Mb. The scaffolds were sorted by length, following clockwise from the longest to the smallest one. Circle (a) shows three layers: i) scaffolds with length and GC content per 1‐kb sliding window; ii) short‐read depth (x, histogram) and long‐read depth (black line) per 10‐kb sliding window; and iii) histogram of heterozygous SNV density (y) per 10‐kb genome window. Circle (b) shows five layers: i) scaffolds with length and gene distribution on scaffold, each gene was displayed by a color representing its GC content; ii) histogram of gene density (z) per 10‐kb genome window; iii) transposable element (TE) distribution on scaffolds with a specific color for each TE family; and iv) horizontal gene transfer (HGT) distribution on scaffold, with color representing GC content of each HGT. Meaning of coded colors in each layer is also given in the middle of each circle

Read‐depth analysis of MaSuRCA, Ra, and Canu assemblies showed a bimodal distribution (Figure S1 – A, B, C). The half‐coverage read‐depth peak on Ra assembly seemed smaller than on MaSuRCA and Canu suggesting that Ra tended to create mostly collapsed haplotype contigs. After purging haplotigs and potential artifacts, the genome assembly sizes were reduced from 47.4–39.7–57.2 Mb (MaSuRCA – Ra – Canu) to 40.9–38.9–42.7 Mb, respectively (Table S4), and the peak at half‐coverage was almost totally absent (Figure S1 – D, E, F). At this stage, Canu showed the best assembly metrics with the longest scaffolds: 1.4 Mb for the largest contig, an N50 length of 292 kb, the smallest number of contigs (i.e., 357), and the lowest number of mismatches (i.e., 300; Table S4). A higher number of reads (long, short, and RNA‐seq) were mapped on the Canu assembly, suggesting a higher efficiency of the Canu software. The genome completeness of the three assemblies remained high with a total BUSCO completeness score superior or equal to 87%. Compared with the initial assembly, the total BUSCO completeness of purged‐haplotigs genome slightly increased in the Canu assembly from 87.2% to 88.1%, while it decreased in the two others, from 90.4% to 89.2% in MaSuRCA and from 89.8% to 87.5% in Ra (Figure 1, Table S4). Besides, the haplotigs purging process allowed a significant increase (+10.8%) in the completeness of single‐copy genes in the Canu genome, while there was a marginal gain in genomes assembled with Ra (+2.0%) and MaSuRCA (+3.7%) (Figure 1, Table S4). In parallel, the completeness of duplicated genes in the Canu genome was strongly decreased (−9.9%) after purging haplotigs, while those were slightly reduced in Ra (−0.3%) and MaSuRCA (−4.9%). The Canu haplotype–purged assembly, which had longer scaffolds and higher completeness, was finally selected as the reference. For the Canu assembly, artifacts (726 kb) were removed by haplotigs purging process. Furthermore, contamination screening detected 74 contigs (total of 1.2 Mb), which had read depth inferior to 100× and showed highest similarity (identity ≥ 70%) to Chordata phylum; therefore, these potential contaminant contigs were filtered out. After removing potential artifacts and contaminations, this final assembly was 41.5 Mb long, with 283 contigs, and an N50 length of 294 kb (Table 1). Figure S2 compares the GC content (peaking at 23%) and read coverage of all contigs. Most of them have a sequencing depth superior to 100× [only two short contigs (i.e., mg287, 11 kb; mg295, 3 kb) with “no‐hit” in the nt database showed a lower depth (68 and 83×)]. One hundred and twenty‐one contigs (covering ca. 29.8 Mb; 71.9% of the genome) contain genomic regions that were identified as belonging to the nematode phylum (identity ≥ 70%; Figure S2). The BUSCO and CEGMA completeness scores for the final assembly were 88.8% and 95.97%, respectively (Table 1). Reads were evenly mapped over most of the scaffolds with a mean coverage of 228× for short reads and 38× for long reads (Figures 2 and S4). The number of heterozygous SNVs varied from 0 to 407 per sliding window, which corresponds to nucleotide divergence ranging from 0% to 5% with a mean value of 1.36 ± 0.78% (Figures 2 and S4).

**Table 1:** Compared statistics of the haplotype‐fused genome assemblies for *M*. *graminicola* obtained in our study (with Canu; Koren et al., 2017) and in Somvanshi et al. (2018)

Assembly features	Canu	Somvanshi et al. (2018)
No. of contigs	283	4,304
Largest contig (bp)	1,433,372	145,493
Total length (bp)	41,549,413	38,184,958
N50	294,907	20,482
N75	185,679	9,797
L50	43	522
L75	78	1,189
GC (%)	23.28	23.49
Mismatches	300	715,992
CEGMA completeness (*n*:248)	C:95.97%	C: 84.27%
BUSCO completeness (*n*:303)	C:88.8% [S:85.8%, D:3.0%]	C: 81.6%

Flow cytometry outputted clearly G0/G1 peaks for each sample and both internal controls (Figure S3). Thanks to the presence of two internal controls, the reference DNA content of one of them could be used as a standard to estimate the DNA content and then the genome size of the other. The calculated genome sizes ranged from 203.9 to 221.6 Mb for *C*. *elegans*, and from 315.8 to 343.3 Mb for *D*. *melanogaster*. These estimates are relatively close to their expected genome sizes (Table S5). The genome size of *C*. *elegans* was closer to *M*. *graminicola* than *D*. *melanogaster*, and therefore, *C. elegans* was used as a standard to calculate the final DNA content of *M*. *graminicola* samples. The total nuclear genome size for four independent measurements of the Mg‐VN18 isolate ranged from 81.5 to 83.8 Mb (average 82.6 Mb), although a fifth estimate was higher and highly suspect (103.9 Mb; Table S5; Figure S3).

### 3.2 Protein‐coding gene annotation

A total number of 10,331 protein‐coding genes were predicted with the Maker2 pipeline, of which 10,284 were selected with AED less than 1 and/or had *Pfam* and InterPro evidence (Table 2). On average, 247.5 protein‐coding genes were thus annotated per Mb. The full genes and their coding sequences (CDS) spanned 75.5% (31.4 Mb) and 28.4% of the total genome length, respectively. Among them, 268 genes showed alternative splice forms, leading to the prediction of 10,631 mRNA with a total length of 32.2 Mb (Table 2). Number of exons per protein‐coding gene varied from 1 to 152 with an average of 11.2 per gene and 8.4 per CDS (Figure S5A). Number of exons per gene was related to gene length (Figure S5B). On average, genes had 4.3 exons per kb, similar to that reported in four cloned genes of *M. graminicola* (on average 4.6 exons/kb in Mg01965, MgM0237, Mg16820, and MgPDI; Chen et al., 2018; Naalden et al., 2018; Tian, Wang, Maria, Qu, & Zheng, 2019; Zhuo et al., 2019). Raw RNA‐seq reads were mapped on 20 eukaryote ortholog genes, which were completely annotated by BUSCO on the *M. graminicola* genome sequence. RNA reads mapped on multiple regions on most of orthologous genes confirming dense distribution of exons in genes of *M. graminicola* (Figure S6). Intronic regions represented 31.9% of the genome, with an average of 10.2 introns per protein‐coding gene. More than 60% of all introns are shorter than 60 nucleotides. Overall, the proportion of canonical splice sites is 94.65% including GT‐AG (92.39%) and CT‐AC (2.26%) for reversed genes. Noncanonical splice sites account for 5.34% consisting of TT‐AG (0.51%), GC‐AG (0.45%), and other minor splice sites (4.39%). The 5′‐UTR and 3′‐UTR spanned 7.6% and 9.7% of the genome, respectively. The GC content of protein‐coding gene was 29.03%, and thus higher than in the whole genome. The length of the 10,631 annotated proteins ranged from ~300 to ~6,000 amino acids (Table 2). The BUSCO completeness of the predicted protein dataset was 86.5% (Table 2). Genes were located in most scaffolds (262 out of 283), and only 21 short scaffolds (<30 kb) did not bear any annotated gene (Figures 2 and S7).

**Table 2:** General characteristics of protein‐coding genes in the *M*. *graminicola* genome reconstructed with Canu (Koren et al., 2017)

Statistics	Protein‐coding gene	mRNA	CDS	Exon	Coding exon	Intron	5′‐UTR	3′‐UTR
Total number	10,284	10,631	10,654	115,769	88,994	105,138	6,756	6,467
Total length (bp)	31,387,211	32,191,507	11,808,477	19,014,544	11,747,208	13,282,101	3,148,471	4,057,596
% genome	75.5	77.5	28.4	45.8	28.2	31.9	7.6	9.7
Mean length (bp)	3,052	3,028	1,110	164	132	126	466	627
Longest length (bp)	38,328	38,328	18,477	5,281	5,821	12,794	17,158	14,255
No. of per protein‐coding gene				11.2	8.4 per CDS	10.2		
No. of per Mb genome	247.51							
GC	29.03%							
BUSCO	C:86.5% [S:81.5%, D:5.0%]							

Abbreviations: CDS, coding sequence; UTR, untranslated region.

### 3.3 Identification and function of horizontal gene transfers

We identified 67 genes encoding 68 proteins that returned an AI > 14, indicating a possible acquisition via HGT from nonmetazoan origin. All these proteins had predicted *pfam* domains, which allowed classifying them in 31 different gene families (Tables 3 and S6). Among them, 54 genes (80.9%) had strong support with AI > 26. A total of 28 genes from six families encode for several plant cell‐wall modification and degradation enzymes such as polygalacturonase, xylanase, arabinase, pectate lyase, expansin‐like proteins, and cellulases. Fourteen genes are possibly involved in nutrient processing (including biosynthesis of vitamins B7, glutamine, and carbohydrate), galactose and sucrose degradation, and transportation of sucrose and sugar moieties. Nine putative HGTs encode for chorismate mutase, isochorismatase, and carboxylesterase that are involved in the detoxification and modulation of plant defense. Other six HGT candidates are related to different pathways such as metabolic processes of nucleosides, amino acids, keto acids, and fatty acids. Two genes encoding peptidase and two others encoding integrase were also identified as HGTs. Other six putative HGTs encode membrane component, carbohydrate‐binding module, thaumatin, unknown protein binding domain, and lysozyme (Table 3). For 92.5% of HGT candidates (62/67), the most similar sequence was of bacterial origin. For the five remaining HGTs, the most similar sequence indicated a potential origin from fungus, archaea, virus and Viridiplantae (Table S6). In addition, a gene encoding cyanate lyase, which contributes to the detoxification process, was detected as an HGT with a low AI score of 4.0. Proteins related to induction of feeding site (candidate acetyltransferase) and biosynthesis of vitamin B1 (VB1 thiD) were present in the *M*. *graminicola* genome. Still, none was detected as putative HGT (AI > 0). The GC content of putative HGTs (Table 3) ranged from 14% to 36% with an average value of 24%. Short‐read coverage over these 67 genes ranged from 100 to 540× (with a mean value of 297×). The value close to the whole sequencing depth suggests putative HGTs were actually part of the *M*. *graminicola* genome. The 67 putative HGTs were located on 47 scaffolds with no apparent hot spot of foreign gene integration (Figures 2 and S7). Besides, average coverage of RNA‐seq reads (at J2 stage) on 67 candidate HGTs was 729×, while the average coverage of these RNA‐seq data on gene set at whole‐genome level was 212×. Among them, putting aside the three genes encoding for putative integrase and glycoprotein (<10×), 64 genes had a RNA‐seq coverage superior to 30, and more interestingly, six of them encoding for putative cellulase, xylanase, and pectinase had a RNA‐seq coverage superior to 1,000×.

**Table 3:** Summary of putative horizontal gene transfers (HGT) in the *Meloidogyne graminicola* genome

General process	Gene/gene family	Function(s)	*N* (AI > 14)	*N* (AI > 26)
Plant cell‐wall degradation	GH28 polygalacturonase	Pectin decoration degradations	3	3
	GH30 xylanase	Xylan degradation	2	2
	GH43 candidate arabinanase	Pectin decoration degradation	1	1
	PL3 pectate lyase	Pectin degradation	10	10
	Expansin‐like proteins	Softening of noncovalent bonds	4	2
	GH5_2 cellulases	Cellulose degradation	8	6
Plant defense manipulation	Candidate isochorismatase	Catalyzes the conversion of isochorismate	1	1
	Chorismate mutase	Conversion of chorismate into SA	1	1
	pnbA carboxylesterase	Hydrolysis of ester and amide bonds	7	6
Nutrient processing	bioB biotin synthase	Vitamin B7 biosynthesis	1	0
	Candidate GS1 glutamine synthetase	Nitrogen assimilation	1	1
	galM candidate galactose mutarotase	Galactose metabolism	1	1
	GH2 β‐galactosidase	Galactose degradation	1	1
	GH32 invertase	Sucrose degradation	2^a^	2^a^
	Sugar transporter (MFS) family	Transport of carbohydrates, organic alcohols, and acids	4	4
	rfaG glycosyltransferase group 1	Catalyzes the transfer of sugar moieties	4	3
Not known	Phosphoribosyltransferase	Nucleoside metabolic process	1	1
	tdk thymidine kinase	Nucleoside metabolic process	1	0
	Candidate L‐threonine aldolase	Cellular amino acid metabolic process	1	1
	Gamma‐glutamylcyclotransferase	Degrade gamma‐glutamylamines to amino acid	1	1
	FAD‐dependent oxidoreductase	Catalyzes D‐amino acids into keto acids	1	1
	HADH	Enzyme involved in fatty acid metabolism	1	0
	DJ‐1/PfpI family cysteine peptidase	Degrade intracellular protein	1	1
	FtsH peptidase	Degrade membrane‐embedded and soluble protein	1	1
	Integrase	Integrates the viral genome into a host chromosome	2	0
	Collagen	Cuticle and basement membrane collagen	1	1
	Phlebovirus glycoprotein G2	Component of Golgi complex membrane	1	0
	Thaumatin‐like protein	Sweet‐tasting protein	1	0
	Domain DUF1772	Unknown	1	1
	Laminin_G_3 family	Carbohydrate‐binding module	1	1
	GH25 Lys1‐like	Bacteria cell‐wall lytic enzyme	1	1

Note

Putative HGTs are classified according to the general process in which they are involved. For each gene family, their supposed function(s) and the number of copies (*N*) are also given. The HGT detection thresholds (Alien Index) are 14 or 26. More details on each gene (i.e., Alien Index, genome location, and accession number) are given in Table S6.

^a^ One gene copy encodes two different proteins (see Table S6).

### 3.4 Diversity and distribution of transposable elements

One hundred and sixteen consensus sequences of repetitive elements were first identified and used as a reference library. This allowed us to annotate 4,513 loci in the genome (16.45% of the genome spanned) among which 575 presented canonical signatures of TEs. Canonical TE annotations spanned 1.08 Mb in total, representing 2.61% of the genome (Table 4). Only canonical TE annotations were then analyzed in detail. DNA transposons were slightly more abundant than retrotransposons, as they respectively covered 1.49% and 1.12% of the genome. Three retrotransposon orders were found, including LINEs (long interspersed nuclear elements), LTRs (long terminal repeats), and TRIMs (terminal repeat retrotransposon in miniatures). The four detected DNA transposons consisted of TIRs (terminal inverted repeats), MITEs (miniature inverted‐repeat transposable elements), Helitrons, and Mavericks (Table 4). Interestingly, the nonautonomous TEs present in the genome (TRIMs, MITEs) accounted for 54.6% of TEs, which corresponded to 1.13% of the total genome assembly (Table 4). TEs were distributed in 195 scaffolds (Figures 2 and S7) with the highest number on scaffolds mg96 (i.e., 22 TEs, density of 1.5 TEs per 10 kb). Two of the three Maverick TEs overlapped with two putative HGT events bearing integrase core domain on scaffolds mg4 and mg32 (Figures 2 and S7).

**Table 4:** Abundance and diversity of transposable elements (TEs) in the *Meloidogyne graminicola* genome

TE family	Number	Total length (bp)	% genome	Minimum length (bp)	Maximum length (bp)
Class I (total)	133	463,595	1.12	–	–
LINEs	5	15,552	0.04	591	5,500
LTRs	26	96,561	0.23	556	8,035
TRIMs	97	340,975	0.82	420	9,959
CLASS_1_LIKE	5	10,507	0.03	417	4,462
Class II (total)	442	621,066	1.49	–	–
TIRs	202	366,687	0.88	387	10,090
MITEs	217	129,768	0.31	258	1,440
Helitrons	16	89,046	0.21	2,842	7,557
Mavericks	3	23,131	0.06	4,441	9,346
CLASS_2_LIKE	4	12,434	0.03	1,506	6,703
Total	575	1,084,661	2.61	–	–

## 4 DISCUSSION

### 4.1 A highly complete and contiguous genome revealed peculiar features in *M. graminicola*

By optimizing DNA extraction methods and utilizing the advantages of long‐read sequencing, the genome assembly of *M*. *graminicola* is here greatly improved compared with the previously published version (Somvanshi et al., 2018). This new genome presents better completeness and a larger genome size with ten times fewer scaffolds. This new assembly yields the second largest N50 length (294 kb) among all *Meloidogyne* genomes publicly available to date (summarized in Susič et al., 2020). The removal of haplotigs and potential contaminants on genome sequence provides a clean genetic material, reducing errors in downstream analyses. Finally, this haplotype‐merged assembly is highly complete regarding CEGMA and BUSCO scores when compared to available RKN genomes (summarized in Koutsovoulos et al., 2019). A higher number of exons per gene (11.2) was detected in *M. graminicola* compared with other PPN species [e.g., ~6 in mitotic RKN (Blanc‐Mathieu et al., 2017); 8.8 in *Globodera rostochiensis* (Akker et al., 2016)]. Frequent noncanonical splice sites (5.34%) were detected in predicted genes of *M. graminicola*, as similarly reported in other nematode species belonging to sister genera [e.g., 3.47% in *G. rostochiensis* (Akker et al., 2016); 4.29% in *Heterodera glycine* (Masonbrink et al., 2019)]. In contrast, a quasiabsence of noncanonical splice sites was reported in RKN species (Akker et al., 2016), but this may be due to restrictive settings during gene annotation in this group. Interestingly, while mainly GC‐AG introns were found as noncanonical in cyst nematode species, several other minor noncanonical splice sites were detected in *M. graminicola*. Such a diversity could be related to an extremely low GC content (23%). In plants and worms, AT content has been demonstrated to represent an important determinant of intron recognition (Aroian et al., 1993; Luehrsen & Walbot, 1994). Notably, nematodes have unique features (e.g., *trans*‐splicing, diverse spliced leader) allowing them to develop specific ways of constructing and altering their genome expression (Barnes et al., 2019; Davis, 1996). Besides, it has been demonstrated that spliceosome mutation of *C. elegans* can lead to recognization of variant sequences at both ends of introns (Aroian et al., 1993). Therefore, we can hypothesize that the *M. graminicola* spliceosome has evolved toward small introns and flexible noncanonical sites recognition, but anyhow further studies are required to support this assumption.

The haploid genome length calculated by *k*‐mer analysis using Illumina reads ranges from 41.1 to 41.6 Mb, which is very similar to the final genome assembly (41.5 Mb). Furthermore, the experimentally measured total DNA content over four replicates ranges from 81.5 to 83.8 Mb, which corresponds to a haploid genome size ranging between 40.7 and 41.9 Mb. These measures suggest our genome assembly is almost complete and corresponds to a haploid genome with merged haplotypes on most genomic regions. This is similar to the facultative sexual *M*. *hapla*, which indicates a canonical sexual diploid genome (Blanc‐Mathieu et al., 2017). The heterozygosity between haplotypes ranges from 1.69% to 1.90%, according to the *k*‐mer analysis and is 1.36% ± 0.78 based on the SNV analysis. In *M. hapla*, meiotic parthenogenesis occurs via terminal fusion (fusion of the terminal products after the two meiotic divisions), which is supposed to homogenize the genome and eventually yield low heterozygosity (Castagnone‐Sereno et al., 2013; Triantaphyllou, 1985). In that perspective, the relatively high heterozygosity in *M. graminicola* is unexpected. It suggests either a different mechanism (i.e., the central fusion of the products of the first division of meiosis) or more frequent outcrossing events. The exact reproductive mode of *M*. *graminicola* thus still needs more investigation, particularly for documenting the process of genome segregation during meiosis.

### 4.2 Evidence of horizontal gene transfers in the *M. graminicola* genome

We identified several robust horizontal gene transfer (HGT) candidates in the *M. graminicola* genome (i.e., 54 genes with AI > 26; Table 3). Many of these genes are predicted to play a role in the degradation of the plant cell wall (44%), which represents a crucial role in parasitism by allowing the migration of parasites in the root tissue. In addition, other HGTs are potentially involved in nutrient biosynthesis and processing, detoxification, and hijack of host plant defenses (Haegeman et al., 2011). A comparison of HGTs discovered in this study with those already known in other RKNs reveals common characteristics, in particular 12 gene families that were phylogenetically supported as HGTs in other PPNs (Table S6). Among them, HGTs encode six plant cell‐wall degradation enzymes, two nutrients processing enzymes, two plant defenses manipulation enzymes, and two unknown proteins, which are all described in details in Appendix S1. In addition, new HGT candidates, not previously described so far in other *Meloidogyne* and with comparably high AI values, are here identified. Specificities of those putative HGTs in *M*. *graminicola* are following summarized by considering the process they are supposedly involved in:

#### 4.2.1 Plant defense manipulation and detoxification

As in other PPNs, candidate HGT genes encoding for chorismate mutase, isochorismate synthase, and cyanate lyases are also found in *M*. *graminicola*. In addition, seven genes encoding carboxylesterases are firstly reported as HGTs in *M. graminicola*. These carboxylesterases might help this parasite to detoxify ester‐containing xenobiotics that are present in phytoalexins secreted by plants in response to nematode infection (Gillet, Bournaud, de Souza, Júnior, & Grossi‐de‐Sa, 2017; Hatfield et al., 2016; Shukla et al., 2017).

#### 4.2.2 Nutrient processing

Some HGTs involved in biosynthesis and process of nutrients have been previously reported in PPNs (Danchin, Guzeeva, Mantelin, Berepiki, & Jones, 2016). Unlike other PPNs, *M*. *graminicola* has more putative HGTs involved in the metabolism linked to the carbohydrate pathways and fewer genes linked to the biosynthesis of vitamins. For instance, only the GH32 gene family related to sucrose degradation has been reported as a HGT in PPNs (Danchin et al., 2016), but we here reveal that 11 *M. graminicola* genes involved in carbohydrate metabolism, galactose degradation, and sugar transport should result from horizontal transfers. Notably, multigenic families encoding for sugar transporters and glycosyltransferase present a high Alien Index (>300) strongly supporting their foreign origin. Interestingly, sugar transporters carry sucrose into the syncytium made by cyst nematodes (*Heterodera* spp.) at the early stage of infection before the establishment of plasmodesmatal connections between the feeding site and the phloem (Zhao et al., 2018). Therefore, such sugar transporters must play a critical role at the early stage of parasitism. In contrast, while nine HGTs involved in the synthesis or salvage of the four vitamins B1, B5, B6, B7 are found in cyst nematode (Craig, Bekal, Niblack, Domier, & Lambert, 2009), *M*. *graminicola* only acquired a single gene encoding vitamin B7 from bacteria. This HGT was not detected in *M*. *incognita*, which, however, acquired HGTs for two other genes encoding vitamins (i.e., B1 and B5; Craig et al., 2009).

#### 4.2.3 Other functions

Novel presumed HGTs with a potential contribution to nematode infection are also detected in *M. graminicola* for the first time: (a) Firstly, *M*. *graminicola* has a candidate GH25 lysozyme likely acquired by HGT and this enzyme could participate in cell division and cell‐wall remodeling in bacteria (Vollmer, Joris, Charlier, & Foster, 2008) and bacteriophages (Fastrez, 1996). Consequently, this gene is suspected of playing a role in the invasion of root tissue (Paganini et al., 2012), but its precise function still remains unknown. (b) Secondly, the HGT candidate with the highest AI (i.e., 370) encodes a protein bearing laminin_G_3 domain belonging to the concanavalin A‐like lectin/glucanases superfamily. This gene is suggested to contribute to cell‐wall degradation process because it acts as a carbohydrate‐binding module and contributes for the hydrolysis activity of arabinofuranosidase (Sakka, Kunitake, Kimura, & Sakka, 2019). (c) Thirdly, in addition to two HGTs putatively involved in the nucleoside metabolic process (candidate phosphoribosyltransferase) and amino acid metabolism (candidate L‐threonine aldolase) previously reported among other PPNs (Danchin et al., 2016), two other genes (encoding candidate gamma‐glutamylcyclotransferase and thymidine kinase) possibly involved in these processes are found for the first time as putative HGTs in *M*. *graminicola*. (d) Fourthly, *M*. *graminicola* has potentially laterally acquired genes for protein degradation and keto acid and fatty acid metabolism. Although there is no clearly defined nematode requirements for these nutriments (i.e., amino acids, fatty acids, keto acids, nucleosides, and acid amins), they are thought to be necessary for PPN development (Goheen, Campbell, & Donald, 2013). Therefore, these HGTs are suspected to contribute to nematodes living inside root tissues. v) Finally, two genes coding for integrase enzymes, which may promote the integration of HGTs into the host chromosome, are also identified as HGTs. Interestingly, these genes are associated with TEs (see Results on “Diversity and distribution of transposable elements”) that potentially created more copies of these genes in the genome. Therefore, they could have themselves contributed to the HGT events observed in *M*. *graminicola*.

Most of these putative HGTs found in the *M*. *graminicola* genome may thus play a crucial role in nematode infection, nutrition requirements, and suppression of plant defenses as already shown in other PPNs (Craig et al., 2009; Danchin et al., 2016; Haegeman et al., 2011). Therefore, these HGTs acquired by *M*. *graminicola* during its evolution have likely contributed to its successful parasitism. Most of these genes, however, have not yet been subjected to functional validation and detailed phylogenetic analysis, so additional studies are still required to identify putative donors and precise the timing of their acquisition and spread.

### 4.3 Diversity and abundance of transposable elements in *M. graminicola*

Transposable elements (TEs) are DNA sequences with the ability to move and to make copies within the genome causing changes in its structure and organization, contributing among other things to the evolution of species (Bonchev & Parisod, 2013; Serrato‐Capuchina & Matute, 2018). More than half of the *M*. *graminicola* TEs are nonautonomous transposons that have lost their transposition machinery. TEs have been annotated in the genomes of other RKNs, including mitotic and meiotic parthenogenetic species (Blanc‐Mathieu et al., 2017; Koutsovoulos et al., 2019). However, as the software version used to annotate the genomes and filters to retrieve canonical TEs was different in each study, the abundance of TEs detected in the facultative meiotic parthenogenetic *M*. *graminicola* is not directly comparable to other species. The TEs load seems to be higher in mitotic parthenogenetic RKNs than in the facultative sexual *M*. *hapla* (Blanc‐Mathieu et al., 2017). In *M*. *enterolobii,* a mitotic parthenogenetic RKN, more nonautonomous TEs were detected (3.12% genome size) than in *M. incognita* and *M*. *javanica* (2.27% and 1.63%, respectively; Koutsovoulos et al., 2019). Considering TE diversity, certain retrotransposon families previously detected in mitotic parthenogenetic RKNs, such as DIRS, SINE, and LARD, are not found in *M*. *graminicola*. Interestingly, the *Cg‐1* gene, whose deletion is associated with resistance‐breaking strains of *M*. *javanica*, has been identified within one transposon (Tm1) belonging to the TIR superfamily suggesting an adaptive impact of TEs on nematode genomes (Gross & Williamson, 2011). Notably, homologs of the Tm1 transposon are also found in the *M*. *graminicola* genome but not in *M*. *hapla*. We also found that two copies of Mavericks bear a HGT encoding DNA integrase, suggesting that some TEs might have been laterally transferred from bacteria to the *M*. *graminicola* genome.

## 5 CONCLUSION AND PERSPECTIVES

This new and more complete genome sequence of *M*. *graminicola* has immediate and important implications for research on the evolutionary biology of this pathogen and on other broader studies of phytoparasitic nematodes. Notably, the high contiguity of the genome presented here enabled us to produce important genetic information, including gene structure and TE/HGT content. This decisive step allows a diversity of investigations at both intra‐ and interspecies levels to decipher geographic origin and diffusion of *M*. *graminicola*, to investigate genome evolution of RKNs associated with their adaptation to different environmental conditions and hosts, and to understand deeper of their evolutionary history.

## CONFLICT OF INTEREST

The authors declare that the research was conducted in the absence of any commercial or financial relationships that could be construed as a potential conflict of interest.

## AUTHOR CONTRIBUTION

**Ngan Thi Phan:** Data curation (lead); Formal analysis (lead); Writing‐original draft (equal). **Julie Orjuela:** Data curation (equal); Formal analysis (equal). **Etienne GJ Danchin:** Conceptualization (equal); Methodology (equal); Writing‐review & editing (equal). **Christophe Klopp:** Data curation (equal); Formal analysis (equal); Writing‐review & editing (equal). **Laetitia Perfus‐Barbeoch:** Investigation (equal). **Djampa Kozlowski:** Formal analysis (equal). **Céline Lopez‐Roques:** Investigation (equal). **Georgios Koutsovoulos:** Formal analysis (equal). **Olivier Bouchez:** Investigation (equal). **Margot Zahm:** Data curation (equal). **Guillaume Besnard:** Conceptualization (equal); Supervision (equal); Writing‐original draft (equal). **Stéphane Bellafiore:** Conceptualization (equal); Funding acquisition (lead); Supervision (equal); Writing‐original draft (equal).

## Supporting information

Supinfo

## Data Availability

All genomic raw sequence reads are accessible as NCBI BioProject PRJNA615787. This Whole Genome Shotgun project has been deposited at DDBJ/ENA/GenBank under the accession JABEBT000000000. The version described in this paper is version JABEBT010000000. Procedural information concerning the genome assembly and analysis presented in this paper can be found at the GitHub repository at https://github.com/PhanNgan/Genome_Assembly_MG
