# Supplementary material for: Genome structure and content of the rice root‐knot nematode (Meloidogyne graminicola)
Source: Ecol Evol. 2020 Sep 13;10(20):11006–21. doi: 10.1002/ece3.6680 (PMC7593179; doi:10.1002/ece3.6680)
Supplement: Supplementary file 1 — Supinfo [file ECE3-10-11006-s001.docx]

**Supporting Information**

**Genome structure and content of the rice root-knot nematode (*Meloidogyne*** ***graminicola*)**

Thi Ngan Phan, Julie Orjuela, Etienne G.J. Danchin, *et al.*

Supporting Information contains:

**Figure S1**. Long-read-coverage analysis of MaSuRCA, Ra and Canu assemblies before (A, B, C) and after (D, E, F) purging haplotigs using long reads

**Figure S2**. Screening of contaminant contigs in the cleaned haplotype-fused genome assembly

**Figure S3**. Cytometry analyses: Relative DNA staining of nuclei at the G0/G1 phase of *M. graminicola*, *Drosophila melanogaster* and *Caenorhabditis elegans*

**Figure S4**. Read-depth and heterozygosity along 277 haplotype-purged scaffolds of the *Meloidogyne graminicola* genome

**Figure S5**. Exon distribution in *Meloidogyne graminicola* genes

**Figure S6**. Per base coverage of raw RNA-seq reads across 20 BUSCO Eukaryote orthologous genes

**Figure S7**. Distribution of genes, transposable elements, and putative horizontal gene transfers on 277 scaffolds of the *Meloidogyne graminicola* genome

**Table S1**. General summary of long and short reads from Oxford Nanopore and Illumina sequencing technology

**Table S2**. Haploid genome size, repeats content and heterozygosity estimated for *M. graminicola* using four *k*-mer values

**Table S3**. Assembly metrics generated by five different methods for the *M. graminicola* genome after polishing and scaffolding processes

**Table S4**. Assembly metrics and BUSCO completeness after purging haplotigs on Canu, Ra and MaSuRCA assemblies

**Table S5**. Total nuclei DNA content of five replicates of *Meloidogyne graminicola* measured by flow cytometry

**Table S6**. Details of putative HGTs detected in the *Meloidogyne graminicola* genome

**Appendix S1**. Summary of putative function(s) of detected HGTs


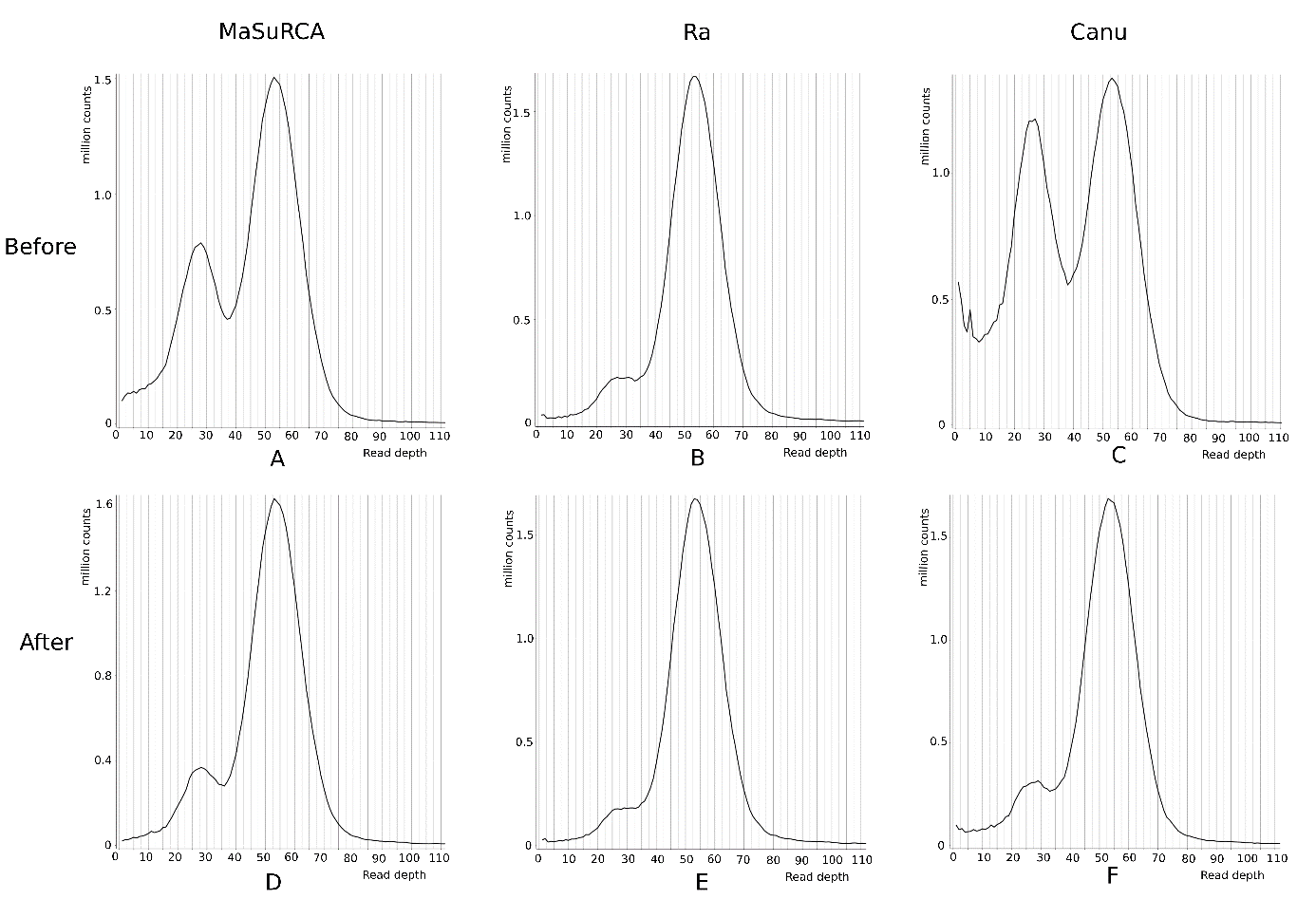


**Figure S1**. Long read-coverage analysis of MaSuRCA, Ra and Canu assemblies before (A, B, C) and after (D, E, F) purging haplotigs using long reads

| 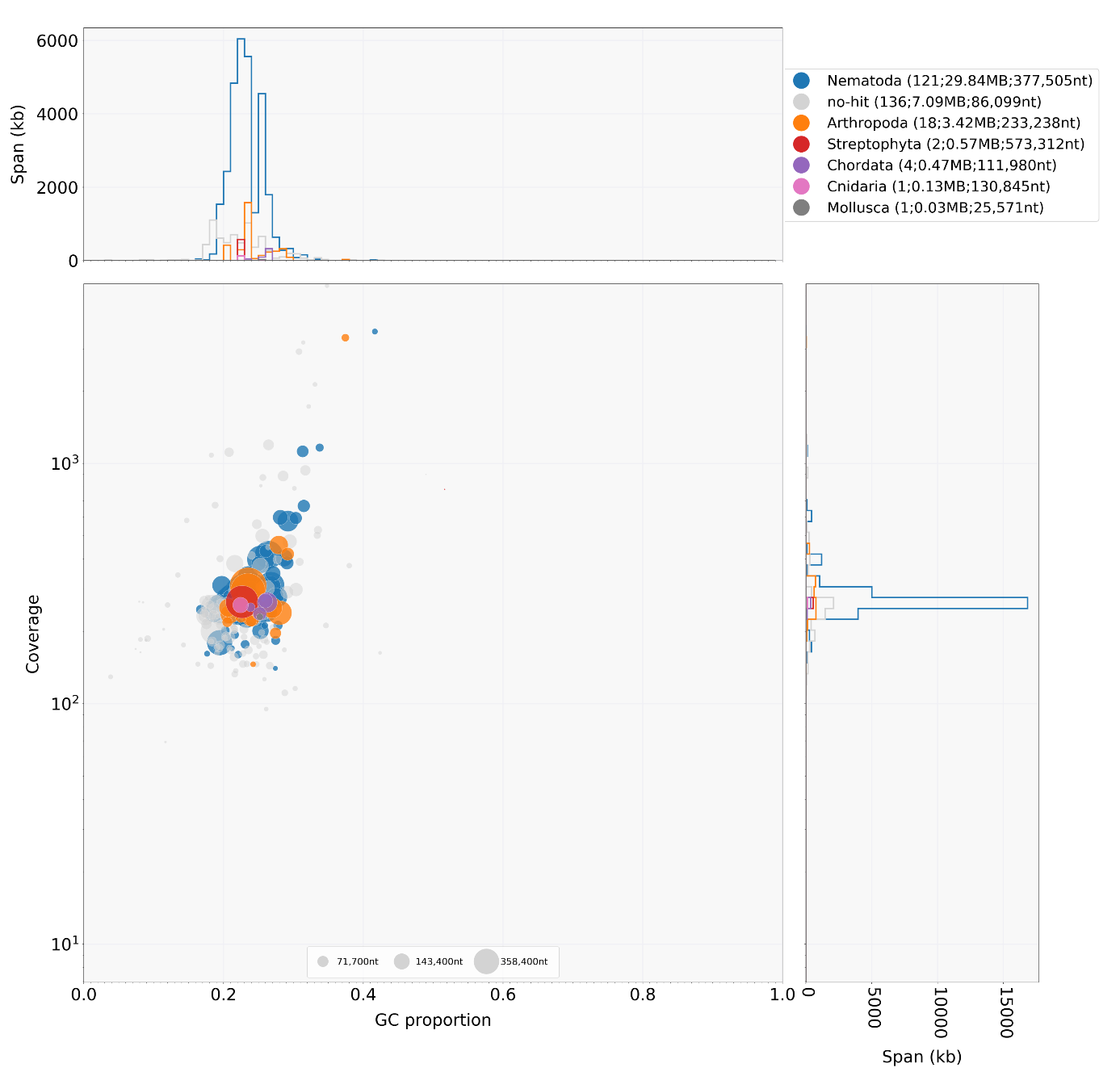 |
| --- |
| **Figure S2**. Screening of contaminant contigs in the cleaned haplotype-fused genome assembly |


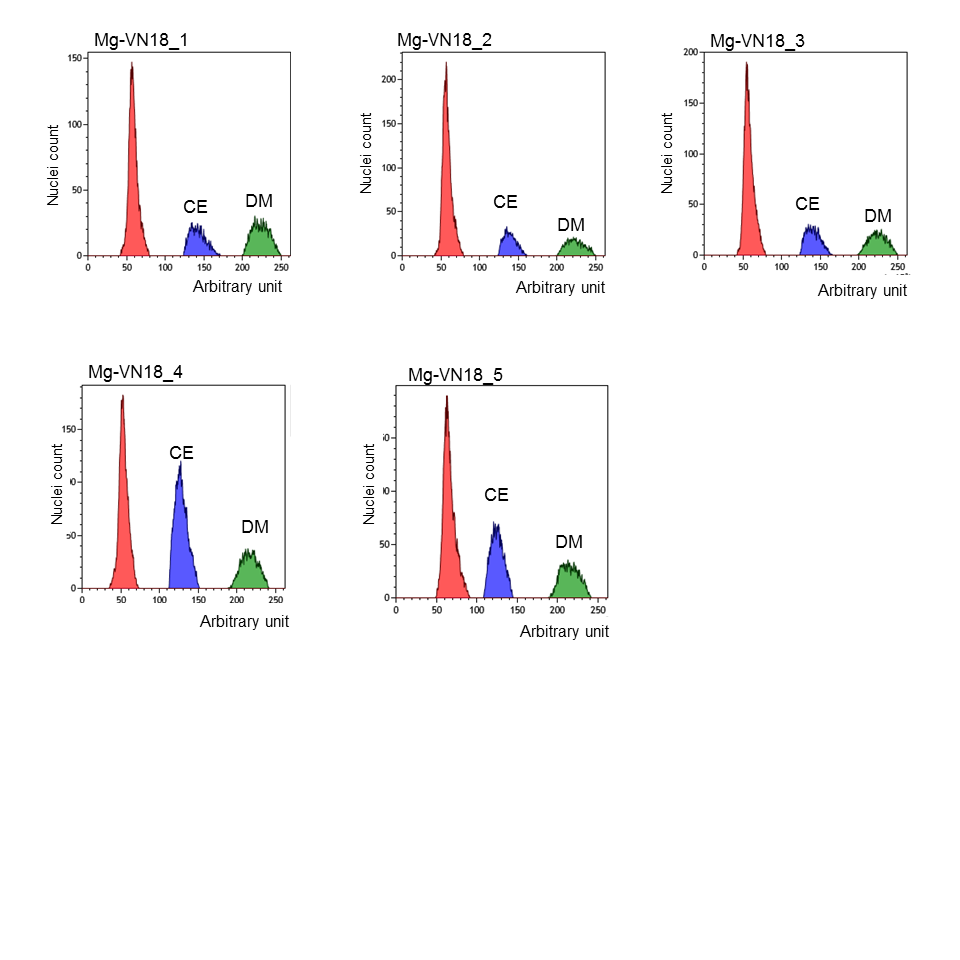


**Figure S3**. Cytometry analyses: Relative DNA staining of nuclei at the G0/G1 phase of *M. graminicola* (Red peak), *Drosophila melanogaster* (DM; green) and *Caenorhabditis* *elegans* (CE; blue). Cytograms showed fluorescence measurement (arbitrary unit) and number of G0/G1 nuclei of five *M. graminicola* isolates of Mg-VN18, mixed with nuclei of *D. melanogaster* (DM; 350 Mb, diploid) and *C. elegans* (CE; 200 Mb, diploid).


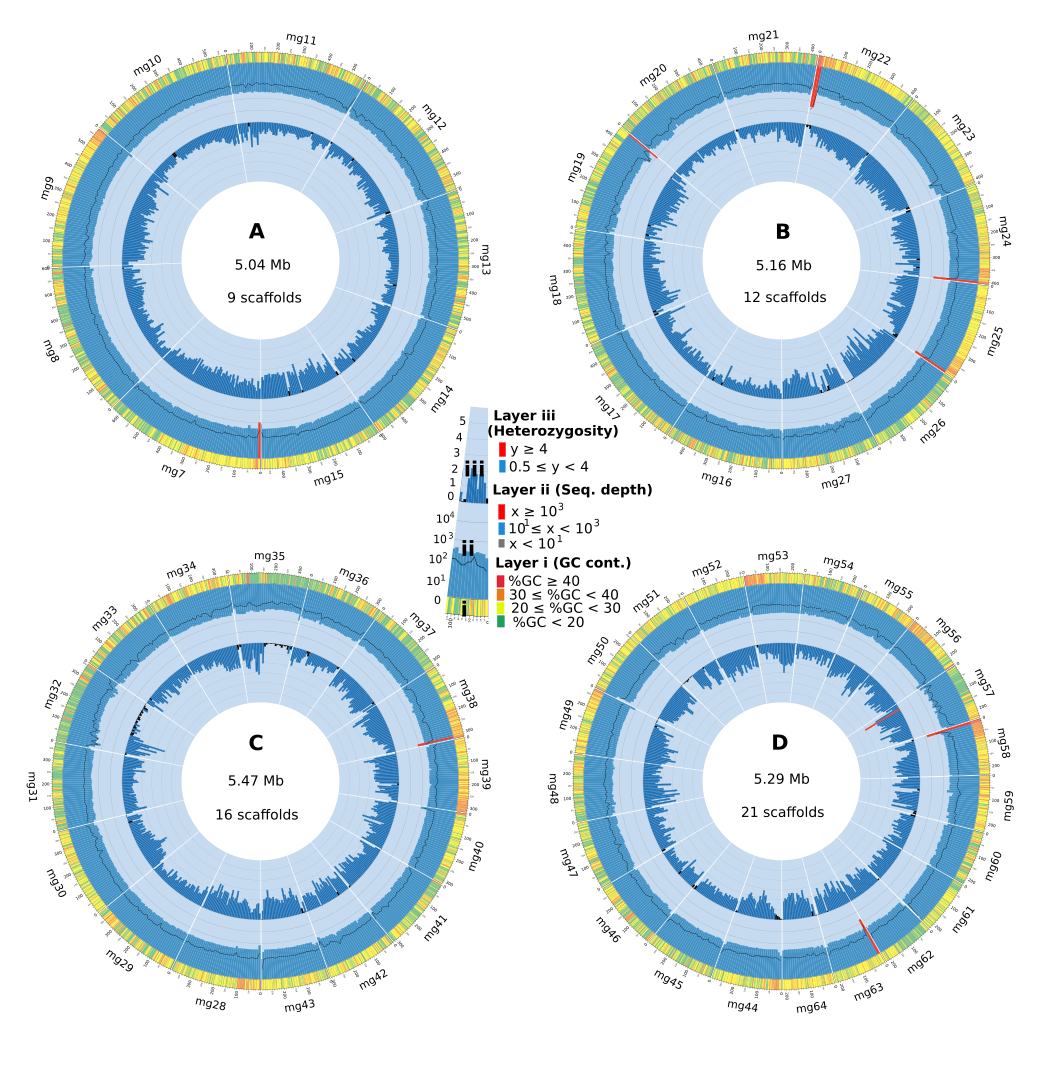


**Figure S4**. Read-depth and heterozygosity along 277 haplotype-purged scaffolds of the *Meloidogyne* *graminicola* genome. Number of scaffolds and total length are indicated in the middle of each circle. The scaffolds are sorted by length following clockwise from the longest to the smallest one. In each circle, three layers represent: (i) the GC content per 1-kb sliding window; (ii) short read-depth (x, histogram) and long read depth (black line) per 10-kb sliding window; and (iii) number of single nucleotide variants (y, histogram) per 10-kb genome window. Meaning of coded colors in each layer is detailed in the middle of the figure.


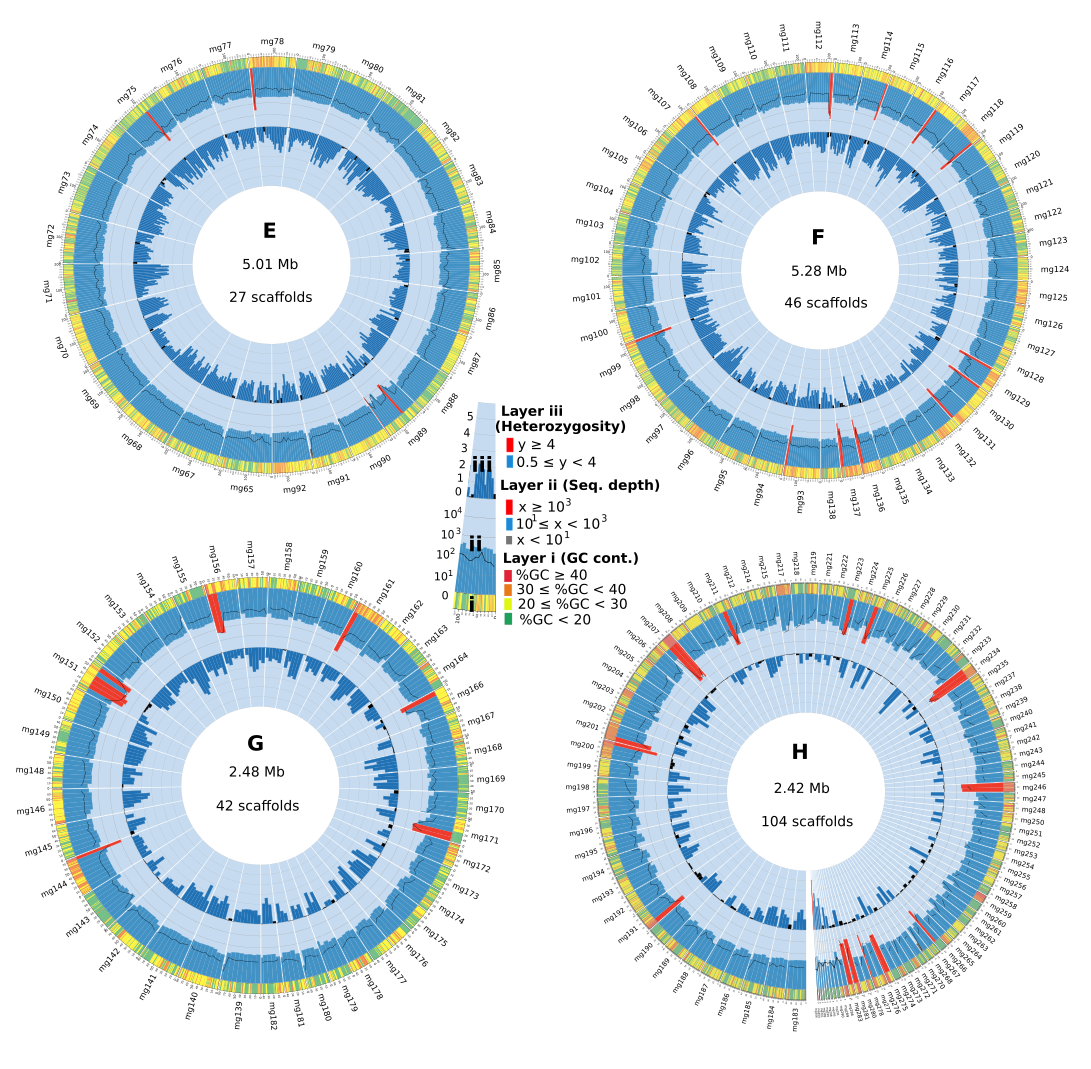


**Figure S4**, end.

|  |  |
| --- | --- |
| (A) | (B) |

**Figure S5**. Exon distribution in *Meloidogyne graminicola* genes: (A) Distribution of the number of exons among all predicted protein coding genes, and (B) number of exons as a function of gene length.

**
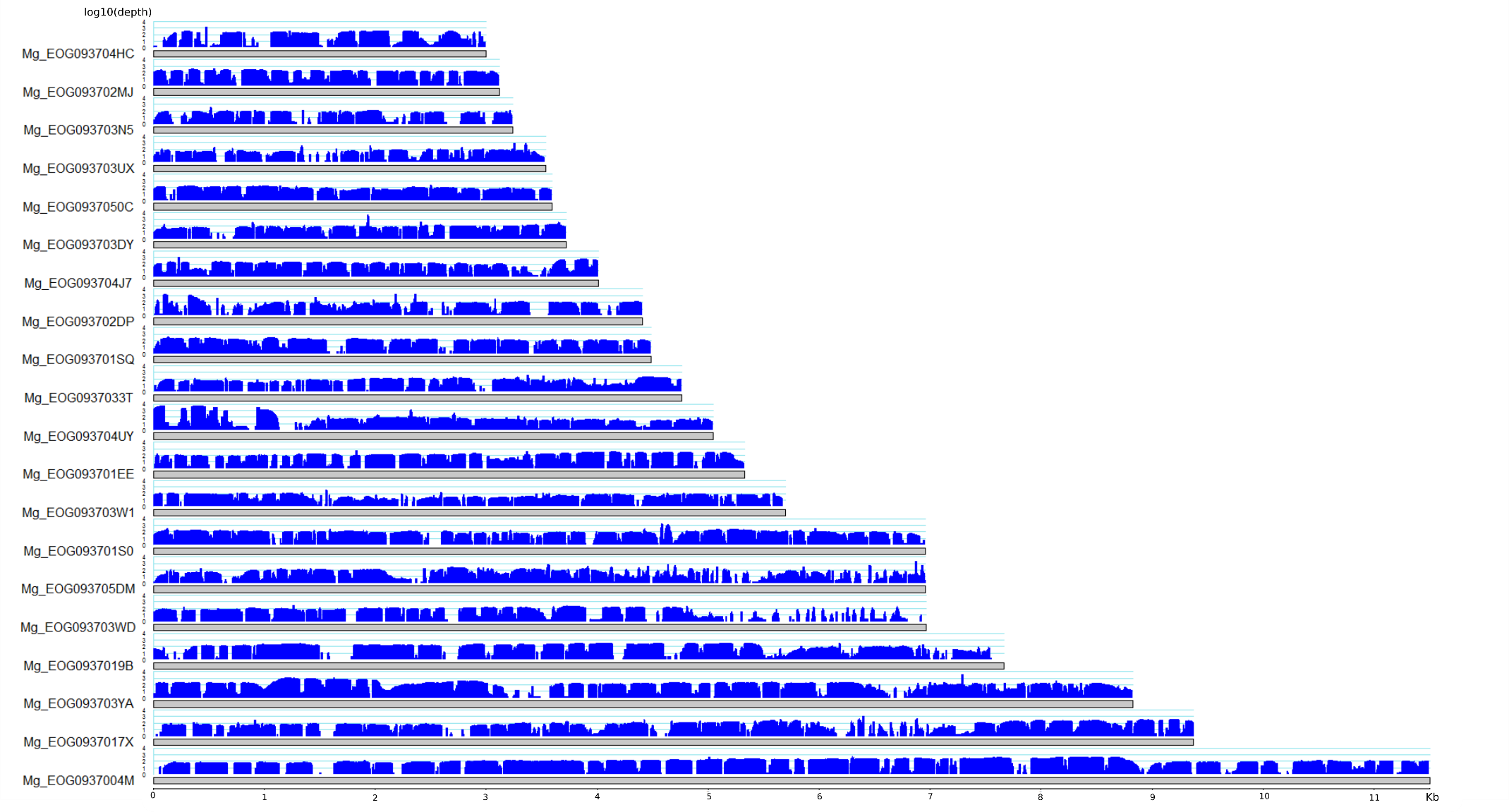
**

**Figure S6**. Per base coverage of raw RNA-seq reads across 20 BUSCO Eukaryote orthologous genes. Each horizontal bar represents a gene. Y-axis indicates the depth of RNA-seq in a log(10) scale


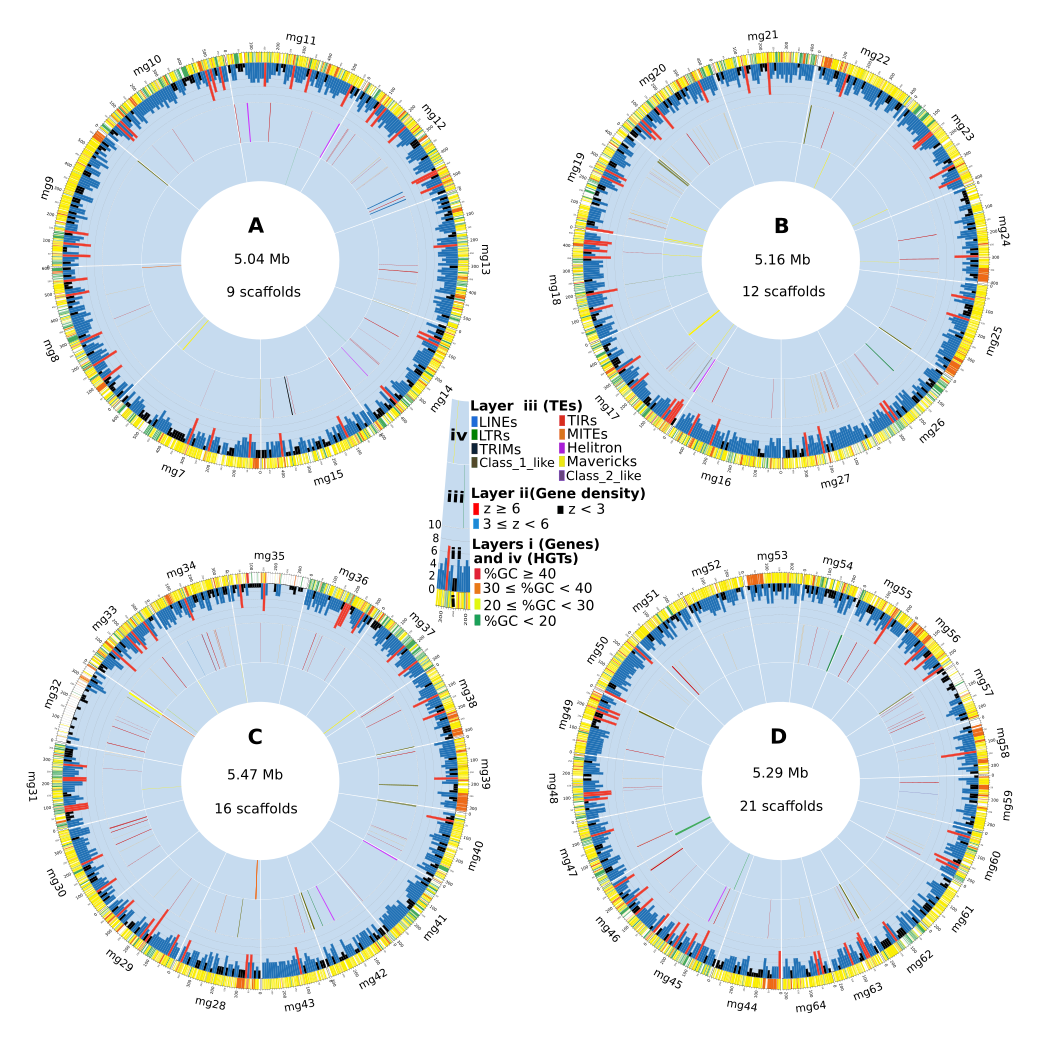


**Figure S7**. Distribution of annotated genes, transposable elements, and putative horizontal gene transfers on 277 scaffolds of the *Meloidogyne graminicola* genome. Number of scaffolds and total length are indicated in the middle of each circle. The scaffolds are sorted by length following clockwise from the longest to the smallest one. In each circle, four layers indicate: (i) location of genes on scaffolds, with a color representing their GC content; (ii) gene density (z, histogram) per 10-kb genome window; (iii) distribution of transposable elements (TEs) on scaffolds, with a specific color for each TE family; (iv) Distribution of horizontal gene transfers (HGTs) on scaffolds, with color representing GC content of each HGT. Meaning of coded colors in each layer is also given in the middle of the figure.


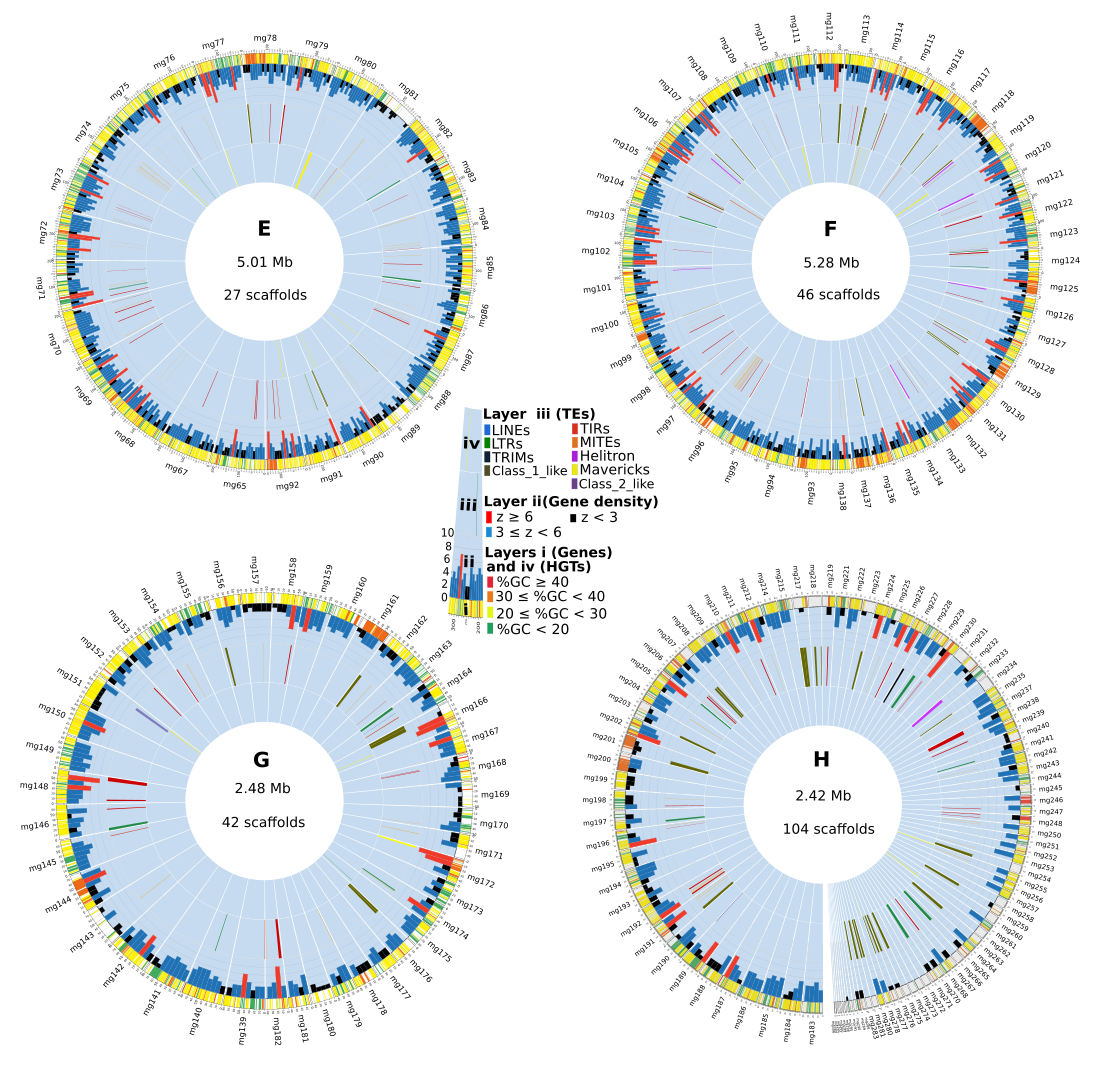


**Figure S7**, end.

**Table S1**. General summary of long and short reads from Oxford Nanopore and Illumina sequencing technology

|  | Nanopore data | |  | Illumina data | |
| --- | --- | --- | --- | --- | --- |
|  | Raw reads | Cleaned reads |  | Raw paired-end reads | Cleaned paired-end reads |
| Number of reads | 869,830 | 727,017 |  | 122 890 657 | 86,991,142 |
| Mean read length | 4,533 | 4,844 |  | 150 | 139 |
| Median read length | 2,558 | 2 ,799 |  | - | - |
| Median read quality | 10.78 | 11.2 |  | 38 | 39 |
| Read N50 length | 8,943 | 9,387 |  | - | - |
| Total bases | 3,943,624,234 | 3,522,271,162 |  | 17 460 298 153 | 11,981,155,793 |
| Estimated coverage* | 94× | 84× |  | 415× | 288× |

* considering assembly genome size of 41.5 Mb

**Table S2**. Haploid genome size, repeats content and heterozygosity estimated for *M. graminicola* using four *k*-mer values with GenomScope (Vulture et al., 2017).

| Genomic feature | *k* value (bp) | | | | |
| --- | --- | --- | --- | --- | --- |
|  | 17 | 21 | 27 | 47 | |
| Haploid genome length (Mb) | 41.1 | 41.4 | 41.3 | 41.6 | |
| Repeat content (Mb) | 19.4 | 11.3 | 7.8 | 7.8 | |
| Heterozygosity (%) | 1.69 | 1.85 | 1.9 | 1.72 | |
|  |  |  | | |  |

**Table S3**. Assembly metrics generated by five different methods after polishing and scaffolding processes

| Process | Assembler | # contigs | Largest contig (bp) | Total length (bp) | N50 (bp) | L50 | GC (%) | CEGMA (%) |
| --- | --- | --- | --- | --- | --- | --- | --- | --- |
| Assembly and polishsing | MaSuRCA | 814 | 864,857 | 47,404,012 | 181,255 | 77 | 23.76 | 95.56 |
|  | Ra | 429 | 498,246 | 39,709,036 | 138,010 | 85 | 23.17 | 93.55 |
|  | Canu | 1,141 | 1,433,372 | 57,182,528 | 186,718 | 77 | 24.37 | 95.97 |
|  | Miniasm | 322 | 2,083,370 | 42,398,816 | 425,868 | 28 | 23.49 | 94.76 |
|  | Flye | 610 | 1,342,289 | 46,902,925 | 197,395 | 56 | 23.59 | 92.74 |
| After scaffolding | MaSuRCA | 808 | 864,857 | 47,404,612 | 189,278 | 76 | 23.76 | 95.56 |
|  | Ra | 420 | 498,246 | 39,709,936 | 146,723 | 82 | 23.17 | 93.55 |
|  | Canu | 1,138 | 1,433,372 | 57,182,828 | 194,506 | 75 | 24.37 | 95.97 |
|  | Miniasm | 322 | 2,083,370 | 42,398,816 | 425,868 | 28 | 23.49 | 94.76 |
|  | Flye | 587 | 1,342,289 | 46,905,225 | 215,369 | 56 | 23.59 | 92.74 |

**Table S4**. Assembly metrics and BUSCO completeness after purging haplotigs on MaSuRCA, Ra, and Canu assemblies

|  | Assemblers | | | | | | | | | | | |
| --- | --- | --- | --- | --- | --- | --- | --- | --- | --- | --- | --- | --- |
|  | MaSuRCA | | |  | | Ra | | |  | Canu | | |
| Metrics | Haplotype-purged  contigs | Haplotigs | Artefacts | |  | Haplotype-purged  contigs | Haplotigs | Artefacts |  | Haplotype-purged  contigs | Haplotigs | Artefacts |
| # contigs | 363 | 419 | 25 | |  | 390 | 27 | 3 |  | 357 | 678 | 103 |
| Largest contig (bp) | 865,251 | 79,346 | 9,809 | |  | 498,246 | 94,725 | 25,679 |  | 1,433,372 | 124,629 | 32,469 |
| Total length (bp) | 40,997,469 | 6,320,255 | 114,560 | |  | 38,894,616 | 765,352 | 49,968 |  | 42,778,169 | 13,677,693 | 726,966 |
| N50 | 221,462 | 20,313 | 4,655 | |  | 152,068 | 31,028 | 25,679 |  | 292,908 | 21,446 | 10,990 |
| N75 | 110,482 | 12,406 | 3,645 | |  | 76,988 | 23,145 | 15,694 |  | 147,791 | 16,457 | 7,536 |
| L50 | 61 | 100 | 9 | |  | 80 | 9 | 1 |  | 45 | 211 | 24 |
| L75 | 126 | 200 | 16 | |  | 171 | 17 | 2 |  | 96 | 393 | 44 |
| GC (%) | 23.55 | 24.9 | 35.65 | |  | 23.21 | 20.96 | 27.23 |  | 23.94 | 25.37 | 31.14 |
| Mismatches (Ns) | 32,744 | 4450 | 0 | |  | 900 | 0 | 0 |  | 300 | 0 | 0 |
| Mapped-short reads (%) | 97.75 |  |  | |  | 98.50 |  |  |  | 98.55 |  |  |
| Mapped-long reads (%) | 87.22 |  |  | |  | 95.64 |  |  |  | 97.06 |  |  |
| Mapped-RNAseq reads (%) | 90.72 |  |  | |  | 89.41 |  |  |  | 90.93 |  |  |
| BUSCO completeness (n: 303)* | S:84.2% D:5.0% | | | |  | S:85.8% D:1.7% | | |  | S:85.1% D:3.0% | | |

* BUSCO analysis was done only on haplotype-purged contigs

**Table S5**. Total nuclei DNA content of five replicates of *Meloidogyne graminicola* (MG; isolate Mg-VN18) measured by flow cytometry (see Figure S3). Nuclei of *Drosophila* *melanogaster* (DM; 350 Mb, diploid) and *Caenorhabditis elegans* (CE; 200 Mb, diploid) were mixed in the same tube as internal controls and served as references to estimate the MG genome size for each replicate.

| Samples | Collecting date | G0/G1 peak value | | | CE genome size based on DM (350Mb) | Genome size based on CE (200 Mb) | |
| --- | --- | --- | --- | --- | --- | --- | --- |
|  |  | CE | DM | MG |  | DM | MG |
| Mg-VN18_1 | 18/04/2019 | 141.2 | 223.0 | 58.2 | 221.6 | 315.8 | 82.4 |
| Mg-VN18_2 | 18/04/2019 | 138.5 | 222.5 | 57.4 | 217.8 | 321.4 | 82.9 |
| Mg-VN18_3 | 18/04/2019 | 140.2 | 223.8 | 57.1 | 219.2 | 319.3 | 81.5 |
| Mg-VN18_4 | 25/02/2019 | 127.3 | 216.1 | 53.3 | 206.2 | 339.5 | 83.8 |
| Mg-VN18_5 | 18/01/2019 | 125.0 | 214.5 | 64.9 | 203.9 | 343.3 | 103.9 |

**Table S6**. Details of putative HGTs detected in the *Meloidogyne graminicola* genome

| Process | Gene/gene family | *Pfam* domain | Functions^1^ | AI^2^ | *M. graminicola* protein accession no | Species with best hit^3^ | *M. inc.*^4^ | *G. ros.*^4^ |
| --- | --- | --- | --- | --- | --- | --- | --- | --- |
| Plant cell wall degradation | GH28 Polygalacturonase | PF00295 Glycosyl hydrolases family 28 | Pectin decorations degradation | 326.9 | Mgra_00000599-RA | (Bac) *Ralstonia pseudosolanacearum* | 351.6 (4) |  |
|  |  |  |  | 325.3 | Mgra_00005314-RA | (Bac) *Ralstonia pseudosolanacearum* |  |  |
|  |  |  |  | 264.4 | Mgra_00010244-RA | (Bac) *Methylibium* sp. CF059 |  |  |
|  | GH30 Xylanase | PF02055; PF17189 Glycosyl hydrolase family 30 TIM-barrel domain; GH30 beta sandwich domain | Xylan degradation | 216 | Mgra_00003019-RA | (Bac) *Clostridium* sp. DL-VIII | 259.49 (6) |  |
|  |  |  |  | 198.2 | Mgra_00008402-RA | (Bac) *Clostridium* sp. DL-VIII |  |  |
|  | GH43 candidate  Arabinase | PF04616 Glycosyl hydrolases family 43 | Pectin decorations degradation | 198.4 | Mgra_00002782-RA | (Bac) *Streptomyces* sp. WAC 00631 | 69.07 (2) |  |
|  | PL3 Pectate lyase | PF03211 Pectate lyase | Pectin degradation | 145.1 | Mgra_00003596-RA | (Bac) *Streptomyces kanasensis* | 137.46 (31) | 137.06 (3) |
|  |  |  |  | 113.9 | Mgra_00002412-RA | (Bac) *Streptomyces viridochromogenes* |  |  |
|  |  |  |  | 91.4 | Mgra_00008269-RA | (Bac) *Streptomyces griseoviridis* |  |  |
|  |  |  |  | 67 | Mgra_00008277-RA | (Bac) *Jonesia quinghaiensis* |  |  |
|  |  |  |  | 66. | Mgra_00010266-RA | (Bac) *Frankia* sp. EAN1pec |  |  |
|  |  |  |  | 64.9 | Mgra_00004469-RA | (Bac) *Frankia* sp. EAN1pec |  |  |
|  |  |  |  | 62.5 | Mgra_00000523-RA | (Bac) *Paraburkholderia monticola* |  |  |
|  |  |  |  | 51.7 | Mgra_00002896-RA | (Bac) *Myxococcales bacterium* |  |  |
|  |  |  |  | 40.9 | Mgra_00004553-RA | (Bac) *Streptomyces sparsogenes* |  |  |
|  |  |  |  | 34.3 | Mgra_00002990-RA | (Bac) *Jonesia quinghaiensis* |  |  |
|  | Expansin-like protein | PF03330 Lytic trans glycolase | Softening of non-covalent bonds | 53.2 | Mgra_00008314-RA | (Bac) *Streptomyces luteoverticillatus* | 86.11 (8) | 29.93 (7) |
|  |  |  |  | 41.7 | Mgra_00009665-RA | (Bac) Streptomycetaceae |  |  |
|  |  |  |  | 15.8 | Mgra_00009039-RA | (Bac) *Streptomyces davaonensis* JCM 4913 |  |  |
|  |  |  |  | 14.2 | Mgra_00007051-RA | (Bac) *Saccharothrix* sp. ST-888 |  |  |
|  | GH5_2 Cellulases | PF00150 Cellulase (glycosyl hydrolase family 5) | Cellulose degradation | 46.2 | Mgra_00008344-RA | (Bac) *Rufibacter tibetensis* | 39.14 (23) | 198.94 (11) |
|  |  |  |  | 39.3 | Mgra_00007596-RA | (Bac) *Salinimicrobium xinjiangense* |  |  |
|  |  |  |  | 32.8 | Mgra_00008505-RA | (Bac) *Hymenobacter terrenus* |  |  |
|  |  |  |  | 32.2 | Mgra_00008504-RA | (Bac) *Rufibacter tibetensis* |  |  |
|  |  |  |  | 29.5 | Mgra_00009334-RA | (Bac) *Flaviramulus basaltis* |  |  |
|  |  |  |  | 28.7 | Mgra_00000930-RA | (Bac) *Aureispira* sp. CCB-QB1 |  |  |
|  |  |  |  | 15.9 | Mgra_00000972-RA | (Bac) *Gramella* sp. SH35 |  |  |
|  |  |  |  | 14.5 | Mgra_00008510-RA | (Bac) *Leeuwenhoekiella polynyae* |  |  |
| Plant defense | Candidate Isochorismatase | PF00857 Isochorismatase family | Catalyses the conversion of isochorismate | 84.0 | Mgra_00000516-RA | (Bac) *Nitratireductor* sp. OM-1 | 91.41 (1) | 66.08 (1) |
|  | Chorismate Mutase | PF01817 Chorismatemutase type II | Conversion of Chorismate into SA | 62.5 | Mgra_00002988-RA | (Bac) *Streptomyces* sp. | 15.02 (1) | 42.36 (2) |
|  | pnbA Carboxylesterase | PF00135 Carboxylesterase family | Esterase | 213.2 | Mgra_00007278-RA | (Bac) *Acidobacteria bacterium* |  |  |
|  |  |  |  | 113.4 | Mgra_00001447-RA | (Fgi) *Saitozyma podzolica* |  |  |
|  |  |  |  | 76.5 | Mgra_00001438-RA | (Bac) *Duganella* sp. |  |  |
|  |  |  |  | 69.6 | Mgra_00004379-RA | (Bac) *Sphingobium* sp. AP49 |  |  |
|  |  |  |  | 58.8 | Mgra_00007277-RA | (Fgi) *Glonium stellatum* |  |  |
|  |  |  |  | 39.4 | Mgra_00007109-RA | (Bac) *Niveispirillum cyanobacteriorum* |  |  |
|  |  |  |  | 22.3 | Mgra_00004380-RA | (Bac) *Acidobacteria bacterium* |  |  |
| Nutrient processing | GH32 invertase | PF00251 Glycosyl hydrolases family 32 N-terminal domain | Sucrose degradation | 328.6 | Mgra_00002716-RA | (Bac) *Sinorhizobium saheli* | 154.42 (1) | 241.26 (11) |
|  |  |  |  | 185.2 | Mgra_00008226-RA | (Bac) *Rhizobium grahamii* |  |  |
|  |  |  |  | 169.7 | Mgra_00008226-RB | (Bac) *Rhizobium grahamii* |  |  |
|  | Candidate GS1 Glutamine Synthetase | PF00120 Glutamine synthetase, catalytic domain | Nitrogen assimilation | 306.6 | Mgra_00008592-RA | (Bac) *Aquamicrobium aerolatum* | 35.59 (4) | 29.24 (1) |
|  | Candidate galactose mutarotase | PF01263 Aldose 1-epimerase | Galactose metabolism | 31.3 | Mgra_00010300-RA | (Bac) *Chelativorans* sp. BNC1 |  |  |
|  | GH2 β-galactosidase | PF02836 Glycosyl hydrolases family 2, TIM barrel domain | Galactose degradation | 293.5 | Mgra_00007323-RA | (Bac) *Ferrovibrio* sp. |  |  |
|  | Sugarporter (MFS transporter family) | PF00083 Sugar (and other) transporter | Transmembrane sugar transporter | 353.9 | Mgra_00007663-RA | (Bac) *Arthrobacter* sp. ZGTC212 |  |  |
|  |  |  |  | 285.6 | Mgra_00003518-RA | (Bac) *Arthrobacter crystallopoietes* |  |  |
|  |  |  |  | 252.1 | Mgra_00004274-RA | (Bac) *Sporolactobacillus* sp. THM19-2 |  |  |
|  |  |  |  | 36.4 | Mgra_00002779-RA | (Bac) *Arthrobacter crystallopoietes* |  |  |
|  | Candidate glycosyl Transferases Group 1 | PF00534 Glycosyl transferases group 1; PF13439 Glycosyltransferase family 4 | Catalyse the transfer of sugar moieties | 307.4 | Mgra_00003366-RA | (Bac) *Cupriavidus gilardii* |  |  |
|  |  |  |  | 238.9 | Mgra_00007025-RA | (Bac) *Proteobacteria bacterium* |  |  |
|  |  |  |  | 29 | Mgra_00008526-RA | (Bac) *Rhizobiales bacterium* |  |  |
|  |  |  |  | 16.6 | Mgra_00008649-RA | (Bac) *Rhizobium leguminosarum* |  |  |
|  | bioB Biotin synthase | PF06968 Biotin and Thiamin Synthesis associated domain; PF04055 Radical SAM superfamily | Vitamin B7 biosynthesis | 22.8 | Mgra_00002952-RA | (Bac) Cardinium endosymbiont  of *Sogatella furcifera* |  |  |
| Suspect function in nematode living | Candidate L-threonine aldolase | PF01212 Beta-eliminating lyase | Amino acid transport and metabolism | 341.2 | Mgra_00005491-RA | (Bac) *Chelativorans* sp. BNC1 | ? (1) | 164.69 (3) |
|  | Gamma-glutamyl cyclo transferase | PF06094 Gamma-glutamyl cyclo transferase, AIG2-like | Gamma-glutamyl amine degradation | 49.6 | Mgra_00008680-RA | (Bac) *Bradyrhizobium diazoefficiens* |  |  |
|  | FAD dependent oxidoreductase | PF01266 FAD dependent oxidoreductase | Keto acid metabolism | 91.5 | Mgra_00007719-RA | (Bac) *Alphaproteobacteria bacterium* 16-39-46 |  |  |
|  | DJ-1/PfpI family cysteine peptidase | PF01965 DJ-1/PfpI family | Degrade intracellular protein | 49.7 | Mgra_00007789-RA | (Bac) *Candidatus Cardinium hertigii* |  |  |
|  | FtsH peptidase | PF01434 Peptidase family M41 | Degrade membrane-protein & soluble protein | 40.1 | Mgra_00007321-RA | (Bac) *Faecalibacterium* sp. An121 |  |  |
|  | HADH | PF00725 3-hydroxyacyl-CoA dehydrogenase; PF02737 C-terminal domain and NAD binding domain | Enzyme involved in fatty acid metabolism | 23.6 | Mgra_00000083-RA | (Bac) *Pararhizobium haloflavum* |  |  |
|  | Phosphoribosyl transferase | PF00156 Phosphoribosyl transferase domain | Nucleoside metabolic process | 200.3 | Mgra_00009552-RA | (Bac) *Chelatococcus* sp. | 202.63 (1) | 198.13 (2) |
|  | tdk Thymidine kinase | PF00265 Thymidine kinase | Nucleoside metabolic process | 25.2 | Mgra_00001990-RA | (Bac) *Candidatus Cardinium hertigii* |  |  |
| Suspect function in cell wall degradation | GH25_Lys1-like | PF01183 Glycosyl hydrolases family 25 | Lysozyme activity | 33.1 | Mgra_00008696-RA | (Arc) Archaeon |  |  |
|  | Laminin_G 3 family | PF13385 Concanavalin A-like lectin/glucanases superfamily; PF05426 Alginate lyase | Carbohydrate-binding module for hydrolysis | 370.9 | Mgra_00004780-RA | (Bac) *Collimonas* sp. OK412 |  |  |
| Support for HGT event | Integrase | PF00665 Integrase core domain | Integrate the foreigner DNA into host genome | 23.3 | Mgra_00004369-RA | (Bac) *Gammaproteobacteria bacterium* |  |  |
|  |  |  |  | 22.6 | Mgra_00001012-RA | (Bac) *Gammaproteobacteria bacterium* |  |  |
| Unknown | Collagen | PF01391 Collagen triple helix repeat (20 copies) | Cuticle and basement membrane collagen | 30.3 | Mgra_00003940-RA | (Bac) *Cellulophaga lytica* |  |  |
|  | Glycoprotein G2 | PF07245 Phlebovirus glycoprotein G2 | Component of Golgi complex membrane | 14.4 | Mgra_00001607-RA | (Viru) Sugarbeet cyst nematode virus 2 |  |  |
|  | [Thaumatin-like protein](https://blast.ncbi.nlm.nih.gov/Blast.cgi#alnHdr_XP_022766133) | PF00314 Thaumatin family | An intensely sweet-tasting protein | 14.2 | Mgra_00000598-RA | (Vird) *Durio zibethinus* |  |  |
|  | Unknown | PF08592 Domain of unknown function (DUF1772) | unknown | 31.1 | Mgra_00000676-RA | (Bac) *Rhodospirillaceae bacterium*  SYSU D60006 |  |  |

^1^ Function of genes was deduced from previous studies performed either on nematodes and/or other groups of organisms (see details, below).

^2^AI =Alien Index.

^3^Bac: Bacteria; Fgi: Fungi; Viru: Virus; Vird: Viridiplantae; Arc: Archaeon.

^4^For each gene/gene family, the highest AI and number of HGTs (AI > 0) found in *Meloidogyne incognita* (*M. inc.*; Abad et al., 2008) and *Globodera rostochiensis* (*G. ros.*; Akker et al., 2016) are given.

**Appendix S1**. Summary of putative function(s) of detected HGTs

Putative function(s) of detected HGTs (Table S6), classified by different processes they are supposedly involved in, are here detailed:

**1. Plant cell wall degradation**. First, putative HGTs encoding cellulases, polygalacturonases, pectate lyases, arabinanase, xylanases, and expansin-like proteins were detected in *M.* *graminicola.* Such enzymes involved in the degradation, modification and softening of the plant cell wall, are usually absent from animal genomes, but have been already reported in a wide range of tylenchomorph PPN species (Danchin, 2011). It was also shown that nematode genes encoding these enzymes also exhibit high similarity to bacterial genes (Danchin, 2011). Such genes may be involved in the root tissue colonization by nematodes, by allowing the degradation of the protective barrier of the plant cell wall, which is constituted mainly of cellulose, hemicelluloses, pectin, and its branched decorations (Malinovsky et al., 2014). It has been already documented that encoded proteins of such HGTs are expressed in the subventral gland cells of nematodes and assist them to penetrate into the roots (Haegeman et al., 2011). More in details, we found in the *M. graminicola* genome:

- a) Eight HGTs encoding cellulases (glycosyl hydrolase family 5). Such genes were the first HGTs to be identified in several cyst nematodes (*G.* *tabacum*, *G.* *rostochiensis*) (Akker et al., 2016; Goellner et al., 2000; Smant et al., 1998), *Pratylenchus* spp. (*P. penetrans*) (Momota et al., 2001), and RKNs (*M. incognita*, *M.* *hapla*) (Opperman et al., 2008; Rosso et al., 1999). Knockout of the gene encoding cellulase (β-1,4, endoglucanases) resulted in the reduction of invasion ability in *G. rostochiensis* (Chen et al., 2005) and the decrease of propagation and dispersal ability in the pine wood nematode *Bursaphelenchus* *xylophilus* (Ma et al., 2011).

- b) Three genes encoding polygalacturonases (glycoside hydrolase GH28 family) and a multigenic family of ten members encoding pectate lyases (PL3s; 10 genes). These enzymes participate in pectin degradation. Such HGTs have been also reported in other RKNs. GH28 polygalacturonases have thus been isolated and biochemically characterized in *M.* *incognita* (Jaubert et al., 2002) and were identified only in RKNs until now. Furthermore, all pectate lyases characterized in plant-parasitic nematodes belong to the polysaccharide lyase family 3, and several copies have been also found [i.e., 22 and 31in *M.* *hapla* and *M. incognita*, respectively (Abad et al., 2008; Opperman et al., 2008)].

- c) Two genes encoding GH30 xylanases (degrading xylan - the major component of hemicelluloses), one gene encoding a candidate GH43 arabinanase (hydrolyzing beta-1,4-galactan in the hairy regions of pectin), and four genes encoding expansin-like proteins (weakener of the non-covalent interactions between cellulose and hemicellulose). Such HGTs were also found in other RKNs (Abad et al., 2008; Opperman et al., 2008).

**2. Plant defense manipulation and detoxification**. We detected in the *M.* *graminicola* genome several HGTs encoding enzymes potentially involved in the parasitism.

First, putative HGTs encoding for chorismate mutase (1 gene), isochorismatase (1 gene), and cyanate lyase (1 gene, AI = 4, not shown in the table) were detected. Such genes were already reported as HGTs in other PPNs, and may play an important role as detoxifying agents or as true suppressors of host defense signaling (Haegeman et al., 2011). They may help obligate endoparasites to modulate host defenses for the duration of their life cycle that lives inside root tissues. Chorismate mutase and isochorismatase, which most closely resemble bacterial enzymes, may reduce the pool of chorismate available for conversion to the plant defense-signaling compound salicylic acid, thus preventing normal activation of host defenses (Wildermuth et al., 2001). It is known that bacterial cyanate lyases can detoxify cyanate, a compound secreted by the plant in response to herbivore attack (Johnson & Anderson, 1987; Jones, 1998). Candidate cyanate lyase has been identified in the genome of *Meloidogyne* spp., which are biotrophic and not herbivorous, but it is possible that the cyanate lyase produced by the nematode has a similar role. However, it should be confirmed by the biochemical function.

In addition, HGTs encoding other enzymes putatively involved in plant defense were here detected for the first time in PPNs. Seven HGTs encoding carboxylesterases were detected in the *M.* *graminicola* genome. Carboxylesterasesare thought to act as a mechanism to detoxify ester-containing xenobiotics, which are toxic properties of phytoalexins, secreted by plants in response to nematode infections (Gillet et al., 2017; Hatfield et al., 2016). In *M. incognita*, one gene (not reported as HGT) encoding a carboxylesterase was upregulated in the compatible interaction with susceptible tomato variety at the latterly stage (13-28 days-after-inoculation) (Shukla et al., 2017).

**3. Nutrition processing**. As a parasite, *M. graminicola* feeds on plant cells by using carbohydrates, amino acids, and vitamins as a source of nutrients for its development. HGTs may have contributed to the optimization of these metabolic processes.

As in other PPNs, we detected HGTs encoding invertases (GH32; 2 loci) and glutamine synthetase (GS1; 1 gene). Invertases (beta-fructofuranosidases) catalyze the hydrolysis of sucrose into fructose and glucose, which can be used by nematodes as a source of energy. A GH32 gene encoding invertase was already phylogenetically supported of bacterial origin in PPNs (Danchin et al., 2016). Similarly, a HGT encoding a glutamine synthetase involved in ammonium assimilation, as part of the nitrogen-fixation pathway in rhizobia, was also reported in PPNs. Furthermore, while nine HGT genes involved in the synthesis or salvage of the four vitamins B1, B5, B6, B7 are found in cyst nematode (Craig et al., 2009), *M.* *graminicola* only acquired a single gene encoding vitamin B7 from bacteria. This HGT was not detected in *M.* *incognita*, which however acquired HGTs for two other genes encoding vitamins (i.e., B1 and B5; Craig et al., 2009).

In addition, several genes potentially involved in the nutrition process were here newly reported as candidate HGTs in the *M.* *graminicola* genome including genes encoding β-galactosidase (GH2; 1 gene), candidate galactose mutarotase (1 gene), glycosyl transferases (4 genes), and sugar transporters (MFS family; 4 genes). β-galactosidase (GH2) catalyzes the hydrolysis of β-galactosides into monosaccharides (*CAZypedia.org*, 2020). In bacteria, galactose mutarotaseis involved in the first step of the galactose metabolism by catalyzing the conversion of β-d-galactose to α-d-galactose (Bouffard et al., 1994; Thoden et al., 2003). Glycosyl transferases of group 1 catalyze the formation of the glycosidic linkage to form a glycoside. These enzymes utilize 'activated' sugar phosphates (including glycogen, fructose-6-phosphate and lipopolysaccharides) as glycosyl donors, and catalyze glycosyl group transfer to a nucleophilic group (Campbell et al., 1997). Sugar porters are responsible for the binding and transport of various carbohydrates, organic alcohols, and acids in a wide range of prokaryotic and eukaryotic organisms, including PPNs, in which sugar transporters are specifically expressed and active in syncytia, indicating a profound role in inter- and intra-cellular transport processes (Hofmann et al., 2009).

**4. Other suspect and unknown function.** The function of a few putative HGTs remains speculative. Here, we briefly summarize the state of current knowledge on these different classes of genes. We detected:

a) One HGT encoding a phosphoribosyl transferase. Such HGTs have been already reported in PPNs, but their functions remained unknown in nematode (Paganini et al., 2012; Scholl et al., 2003). Phosphoribosyl transferase catalyzes the displacement of the alpha-1'-pyrophosphate of 5-phosphoribosyl-alpha 1-pyrophosphate by a nitrogen-containing nucleophile (https://pfam.xfam.org). This domain is found in a range of diverse phosphoribosyl transferase enzymes and regulatory proteins of the nucleotide synthesis and salvage pathways (Sinha & Smith, 2001).

b) One HGT encoding a L-threonine aldolase (TA). TAs represent a family of homologous pyridoxal 5’-phosphate-dependent enzymes found in bacteria and fungi, and catalyze the reversible cleavage of several l-3-hydroxy-α-amino acids (di Salvo et al., 2014). HGTs encoding L-threonine aldolases have been already detected in *Meloidogyne* (Scholl et al., 2003), and these genes (as well as those encoding glutamine synthetase and *nodL*) were probably acquired from a *Mesorhizobium* species (Scholl et al., 2003).

c) One HGT encoding gamma-glutamylamine cyclotransferase. This ubiquitous enzyme is found in bacteria, plants, and metazoans from *Dictyostelium* through to humans. It catalyzes the conversion of epsilon-(L-gamma-glutamyl)-L-lysine to free lysine and 5-oxo-L-proline as well as the release of free amines and the formation of 5-oxo-L-proline from a variety of other L-gamma-glutamylamines (Oakley et al., 2010). The free lysine and/or amines would be utilized by nematodes for their living.

d) One HGT encoding FAD dependent oxidoreductase. This *M. graminicola* gene shows 31% identity with the D-aspartate oxidase (DDO) gene and 25% identity with D-amino-acid oxidase (DAO) genes of *C. elegans* (Katane et al., 2010). DAO catalyzes the oxidation of neutral and basic D-amino acids into their corresponding keto acids while DDO, structurally related to DAO, catalyzes the same reaction but is active only toward dicarboxylic D-amino acids. In higher animals, DAO and DDO regulate endogenous d-Ser and d-Asp levels, respectively, as well as mediate the elimination of accumulated exogenous d-amino acids in various organs (Katane et al., 2010).

e) One HGT for PfpI. The function of this gene is unclear in nematodes, however, it was characterized in bacteria as endopeptidase which degrades intracellular proteins to free acid amines (Halio et al., 1996; Zhan et al., 2014).

f) One HGT for FtsH, an ATP-dependent integral membrane protease. It plays a crucial role in quality control of integral membrane proteins by degrading unneeded or damaged membrane proteins, but it also targets cytoplasmic soluble proteins (Bieniossek et al., 2006).

g) One HGT for 3-hydroxyacyl-CoA dehydrogenase (EC) (HCDH). This enzyme is involved in fatty acid metabolism by catalyzing the reduction of 3-hydroxyacyl-CoA to 3-oxoacyl-CoA. There are two major regions of similarities in protein sequences of the HCDH family, the first one located in the N-terminal, corresponds to the NAD-binding site, while the second one is located in the center of the sequence, which represents the C-terminal domain (Birktoft et al., 1987).

h) One HGT for Thymidine kinase. This ubiquitous enzyme catalyzes the ATP-dependent phosphorylation of thymidine, and has been already characterized in *C. elegans* (Skovgaard & Munch-Petersen, 2006).

i) Two genes coding for integrase enzymes. Integrases may be essential for the integration of HGTs into the host chromosome, and are also identified as HGTs in *M. graminicola*. Interestingly, these genes were associated with TEs that potentially created more copies of these genes in the genome. Therefore, they could have themselves contributed to the HGT events observed in *M.* *graminicola*

j) One HGT for collagen triple helix repeat. Collagens are generally extracellular structural proteins involved in formation of connective tissue structure. The alignment contains 20 copies of the G-X-Y repeat that forms a triple helix. The collagen genes of nematodes encode proteins that have a diverse range of functions. Among their most abundant products are the cuticular collagens, which include about 80% of the proteins present in the nematode cuticle (Fetterer & Rhoads, 1993).

k) One HGT for glycoprotein G2. Glycoprotein G2 is component of the viral envelop in the Bunyaviruses family (Andersson & Pettersson, 1998). This gene in *M.* *graminicola* shows 40% identity with a glycoprotein precursor that has been found in the sugar beet cyst nematode virus (Lin et al., 2018).

l) One HGT encoding a thaumatin-like protein (TLP). TLPs are polypeptides of about 200 residues synthesized by plants in response to fungal infection (Zhang et al., 2018). The antifungal function of this gene was also proposed in *Schistocerca* *gregaria* and *C.* *elegans* (Brandazza et al., 2004; Dierking et al., 2016), however, the detailed mechanism is not yet completely understood.

**References**

Abad, P., Gouzy, J., Aury, J.-M., Castagnone-Sereno, P., Danchin, E. G. J., Deleury, E., Perfus-Barbeoch, L., Anthouard, V., Artiguenave, F., Blok, V. C., Caillaud, M.-C., Coutinho, P. M., Dasilva, C., De Luca, F., Deau, F., Esquibet, M., Flutre, T., Goldstone, J. V., Hamamouch, N., … Wincker, P. (2008). Genome sequence of the metazoan plant-parasitic nematode *Meloidogyne incognita*. *Nature Biotechnology*, *26*, 909. https://doi.org/10.1038/nbt.1482

Akker, S. E. den, Laetsch, D. R., Thorpe, P., Lilley, C. J., Danchin, E. G. J., Rocha, M. D., Rancurel, C., Holroyd, N. E., Cotton, J. A., Szitenberg, A., Grenier, E., Montarry, J., Mimee, B., Duceppe, M.-O., Boyes, I., Marvin, J. M. C., Jones, L. M., Yusup, H. B., Lafond-Lapalme, J., … Jones, J. T. (2016). The genome of the yellow potato cyst nematode, *Globodera rostochiensis* , reveals insights into the basis of parasitism and virulence. *Genome Biology*, *17*(1), 1–23. https://doi.org/10.1186/s13059-016-0985-1

Andersson, A. M., & Pettersson, R. F. (1998). Targeting of a short peptide derived from the cytoplasmic tail of the G1 membrane glycoprotein of *Uukuniemi virus* (Bunyaviridae) to the Golgi complex. *Journal of Virology*, *72*(12), 9585–9596.

Bieniossek, C., Schalch, T., Bumann, M., Meister, M., Meier, R., & Baumann, U. (2006). The molecular architecture of the metalloprotease FtsH. *Proceedings of the National Academy of Sciences of the United States of America*, *103*(9), 3066–3071. https://doi.org/10.1073/pnas.0600031103

Birktoft, J. J., Holden, H. M., Hamlin, R., Xuong, N. H., & Banaszak, L. J. (1987). Structure of L-3-hydroxyacyl-coenzyme A dehydrogenase: preliminary chain tracing at 2.8-A resolution. *Proceedings of the National Academy of Sciences of the United States of America*, *84*(23), 8262–8266. https://doi.org/10.1073/pnas.84.23.8262

Bouffard, G. G., Rudd, K. E., & Adhya, S. L. (1994). Dependence of lactose metabolism upon mutarotase encoded in the gal operon in *Escherichia coli*. *Journal of Molecular Biology*, *244*(3), 269–278. https://doi.org/10.1006/jmbi.1994.1728

Brandazza, A., Angeli, S., Tegoni, M., Cambillau, C., & Pelosi, P. (2004). Plant stress proteins of the thaumatin-like family discovered in animals. *FEBS Letters*, *572*(1–3), 3–7. https://doi.org/10.1016/j.febslet.2004.07.003

Campbell, J., Davies, G., Bulone, V., & Henrissat, B. (1997). A classification of nucleotide-diphospho-sugar glycosyltransferases based on amino acid sequence similarities. *The Biochemical Journal*, *326*(Pt 3), 929–939. https://doi.org/10.1042/bj3260929u

*CAZypedia.org*. (2020, January 23). Glycoside Hydrolase Family 2. CAZypedia.org

Chen, Q., Rehman, S., Smant, G., & Jones, J. T. (2005). Functional analysis of pathogenicity proteins of the potato cyst nematode *Globodera rostochiensis* using RNAi. *Molecular Plant-Microbe Interactions*, *18*(7), 621–625. https://doi.org/10.1094/MPMI-18-0621

Craig, J. P., Bekal, S., Niblack, T., Domier, L., & Lambert, K. N. (2009). Evidence for horizontally transferred genes involved in the biosynthesis of vitamin B1, B5, and B7 in *Heterodera* *glycines*. *Journal of Nematology*, *41*(4), 281–290.

Danchin, É. G. J. (2011). What nematode genomes tell us about the importance of horizontal gene transfers in the evolutionary history of animals. *Mobile Genetic Elements*, *1*(4), 269–273. https://doi.org/10.4161/mge.18776

Danchin, G. J. E., Guzeeva, A. E., Mantelin, S., Berepiki, A., & Jones, T. J. (2016). Horizontal gene transfer from bacteria has enabled the plant-parasitic nematode *Globodera pallida* to feed on host-derived sucrose. *Molecular Biology and Evolution*, *33*(6), 1571–1579. https://doi.org/10.1093/molbev/msw041

di Salvo, M. L., Remesh, S. G., Vivoli, M., Ghatge, M. S., Paiardini, A., D’Aguanno, S., Safo, M. K., & Contestabile, R. (2014). On the catalytic mechanism and stereospecificity of *Escherichia* *coli* L-threonine aldolase. *The FEBS Journal*, *281*(1), 129–145. https://doi.org/10.1111/febs.12581

Dierking, K., Yang, W., & Schulenburg, H. (2016). Antimicrobial effectors in the nematode *Caenorhabditis* *elegans*: an outgroup to the Arthropoda. *Philosophical Transactions of the Royal Society B: Biological Sciences*, *371*(1695). https://doi.org/10.1098/rstb.2015.0299

Fetterer, R. H., & Rhoads, M. L. (1993). Biochemistry of the nematode cuticle: relevance to parasitic nematodes of livestock. *Veterinary Parasitology*, *46*(1–4), 103–111. https://doi.org/10.1016/0304-4017(93)90051-n

Gillet, F.-X., Bournaud, C., Antonino de Souza Júnior, J. D., & Grossi-de-Sa, M. F. (2017). Plant-parasitic nematodes: towards understanding molecular players in stress responses. *Annals of Botany*, *119*(5), 775–789. https://doi.org/10.1093/aob/mcw260

Goellner, M., Smant, G., De Boer, J. M., Baum, T. J., & Davis, E. L. (2000). Isolation of beta-1,4-endoglucanase genes from *Globodera tabacum* and their expression during parasitism. *Journal of Nematology*, *32*(2), 154–165.

Haegeman, A., Jones, J. T., & Danchin, E. G. J. (2011). Horizontal gene transfer in nematodes: A catalyst for plant parasitism? *Molecular Plant-Microbe Interactions*, *24*(8), 879–887. https://doi.org/10.1094/MPMI-03-11-0055

Halio, S. B., Blumentals, I. I., Short, S. A., Merrill, B. M., & Kelly, R. M. (1996). Sequence, expression in *Escherichia coli*, and analysis of the gene encoding a novel intracellular protease (PfpI) from the hyperthermophilic archaeon *Pyrococcus furiosus*. *Journal of Bacteriology*, *178*(9), 2605–2612.

Hatfield, M. J., Umans, R. A., Hyatt, J. L., Edwards, C. C., Wierdl, M., Tsurkan, L., Taylor, M. R., & Potter, P. M. (2016). Carboxylesterases: General detoxifying enzymes. *Chemico-Biological Interactions*, *259*(Pt B), 327–331. https://doi.org/10.1016/j.cbi.2016.02.011

Hofmann, J., Hess, P. H., Szakasits, D., Blöchl, A., Wieczorek, K., Daxböck-Horvath, S., Bohlmann, H., van Bel, A. J. E., & Grundler, F. M. W. (2009). Diversity and activity of sugar transporters in nematode-induced root syncytia. *Journal of Experimental Botany*, *60*(11), 3085–3095. https://doi.org/10.1093/jxb/erp138

Jaubert, S., Laffaire, J.-B., Abad, P., & Rosso, M.-N. (2002). A polygalacturonase of animal origin isolated from the root-knot nematode *Meloidogyne incognita*. *FEBS Letters*, *522*(1–3), 109–112. https://doi.org/10.1016/s0014-5793(02)02906-x

Johnson, W. V., & Anderson, P. M. (1987). Bicarbonate is a recycling substrate for cyanase. *The Journal of Biological Chemistry*, *262*(19), 9021–9025.

Jones, D. A. (1998). Why are so many food plants cyanogenic? *Phytochemistry*, *47*(2), 155–162. https://doi.org/10.1016/s0031-9422(97)00425-1

Katane, M., Saitoh, Y., Seida, Y., Sekine, M., Furuchi, T., & Homma, H. (2010). Comparative characterization of three D-Aspartate oxidases and one D-amino acid oxidase from *Caenorhabditis* *elegans*. *Chemistry & Biodiversity*, *7*(6), 1424–1434. https://doi.org/10.1002/cbdv.200900294

Lin, J., Ye, R., Thekke-Veetil, T., Staton, M. E., Arelli, P. R., Bernard, E. C., Hewezi, T., Domier, L. L., & Hajimorad, M. R. (2018). A novel picornavirus-like genome from transcriptome sequencing of sugar beet cyst nematode represents a new putative genus. *The Journal of General Virology*, *99*(10), 1418–1424. https://doi.org/10.1099/jgv.0.001139

Ma, H. B., Lu, Q., Liang, J., & Zhang, X. Y. (2011). Functional analysis of the cellulose gene of the pine wood nematode, *Bursaphelenchus xylophilus*, using RNA interference. *Genetics and Molecular Research*, *10*(3), 1931–1941. https://doi.org/10.4238/vol10-3gmr1367

Malinovsky, F. G., Fangel, J. U., & Willats, W. G. T. (2014). The role of the cell wall in plant immunity. *Frontiers in Plant Science*, *5*. https://doi.org/10.3389/fpls.2014.00178

Momota, Y., Uehara, T., & Kushida, A. (2001). PCR-based cloning of two β-1,4-endoglucanases from the root-lesion nematode *Pratylenchus penetrans*. *Nematology*, *3*(4), 335–341. https://doi.org/10.1163/156854101317020259

Oakley, A. J., Coggan, M., & Board, P. G. (2010). Identification and characterization of gamma-glutamylamine cyclotransferase, an enzyme responsible for gamma-glutamyl-epsilon-lysine catabolism. *The Journal of Biological Chemistry*, *285*(13), 9642–9648. https://doi.org/10.1074/jbc.M109.082099

Opperman, C. H., Bird, D. M., Williamson, V. M., Rokhsar, D. S., Burke, M., Cohn, J., Cromer, J., Diener, S., Gajan, J., Graham, S., Houfek, T. D., Liu, Q., Mitros, T., Schaff, J., Schaffer, R., Scholl, E., Sosinski, B. R., Thomas, V. P., & Windham, E. (2008). Sequence and genetic map of *Meloidogyne hapla*: A compact nematode genome for plant parasitism. *Proceedings of the National Academy of Sciences*, *105*(39), 14802–14807. https://doi.org/10.1073/pnas.0805946105

Paganini, J., Campan-Fournier, A., Da Rocha, M., Gouret, P., Pontarotti, P., Wajnberg, E., Abad, P., & Danchin, E. G. J. (2012). Contribution of lateral gene transfers to the genome composition and parasitic ability of root-knot nematodes. *PLoS ONE*, *7*(11), e50875. https://doi.org/10.1371/journal.pone.0050875

Rosso, M. N., Favery, B., Piotte, C., Arthaud, L., De Boer, J. M., Hussey, R. S., Bakker, J., Baum, T. J., & Abad, P. (1999). Isolation of a cDNA encoding a beta-1,4-endoglucanase in the root-knot nematode *Meloidogyne incognita* and expression analysis during plant parasitism. *Molecular Plant-Microbe Interactions*, *12*(7), 585–591. https://doi.org/10.1094/MPMI.1999.12.7.585

Scholl, E. H., Thorne, J. L., McCarter, J. P., & Bird, D. M. (2003). Horizontally transferred genes in plant-parasitic nematodes: a high-throughput genomic approach. *Genome Biology*, *4*(6), R39. https://doi.org/10.1186/gb-2003-4-6-r39

Shukla, N., Yadav, R., Kaur, P., Rasmussen, S., Goel, S., Agarwal, M., Jagannath, A., Gupta, R., & Kumar, A. (2017). Transcriptome analysis of root‐knot nematode (*Meloidogyne incognita*)‐infected tomato (*Solanum lycopersicum*) roots reveals complex gene expression profiles and metabolic networks of both host and nematode during susceptible and resistance responses. *Molecular Plant Pathology*, *19*(3), 615–633. https://doi.org/10.1111/mpp.12547

Sinha, S. C., & Smith, J. L. (2001). The PRT protein family. *Current Opinion in Structural Biology*, *11*(6), 733–739. https://doi.org/10.1016/S0959-440X(01)00274-3

Skovgaard, T., & Munch-Petersen, B. (2006). Purification and characterization of wild-type and mutant TK1 type kinases from *Caenorhabditis elegans*. *Nucleosides, Nucleotides & Nucleic Acids*, *25*(9–11), 1165–1169. https://doi.org/10.1080/15257770600894410

Smant, G., Stokkermans, J. P., Yan, Y., de Boer, J. M., Baum, T. J., Wang, X., Hussey, R. S., Gommers, F. J., Henrissat, B., Davis, E. L., Helder, J., Schots, A., & Bakker, J. (1998). Endogenous cellulases in animals: isolation of beta-1, 4-endoglucanase genes from two species of plant-parasitic cyst nematodes. *Proceedings of the National Academy of Sciences of the United States of America*, *95*(9), 4906–4911. https://doi.org/10.1073/pnas.95.9.4906

Thoden, J. B., Kim, J., Raushel, F. M., & Holden, H. M. (2003). The catalytic mechanism of galactose mutarotase. *Protein Science*, *12*(5), 1051–1059.

Vurture, G. W., Sedlazeck, F. J., Nattestad, M., Underwood, C. J., Fang, H., Gurtowski, J., & Schatz, M. C. (2017). GenomeScope: fast reference-free genome profiling from short reads. *Bioinformatics*, *33*(14), 2202–2204. https://doi.org/10.1093/bioinformatics/btx153

Wildermuth, M. C., Dewdney, J., Wu, G., & Ausubel, F. M. (2001). Isochorismate synthase is required to synthesize salicylic acid for plant defence. *Nature*, *414*(6863), 562–565. https://doi.org/10.1038/35107108

Zhan, D., Bai, A., Yu, L., Han, W., & Feng, Y. (2014). Characterization of the PH1704 protease from *Pyrococcus* *horikoshii* OT3 and the critical functions of Tyr120. *PLoS ONE*, *9*(9), e103902. https://doi.org/10.1371/journal.pone.0103902

Zhang, J., Wang, F., Liang, F., Zhang, Y., Ma, L., Wang, H., & Liu, D. (2018). Functional analysis of a pathogenesis-related thaumatin-like protein gene TaLr35PR5 from wheat induced by leaf rust fungus. *BMC Plant Biology*, *18*. https://doi.org/10.1186/s12870-018-1297-2
